# Parasite-specific essential bromodomain protein TgBDP4 is a key epigenetic reader and a potential drug target for the parasite *Toxoplasma gondii*

**DOI:** 10.1016/j.jbc.2026.113160

**Published:** 2026-05-14

**Authors:** Rajkumar Gurupwar, Chitti Raju Khandavalli, Abhijit S. Deshmukh

**Affiliations:** 1 Molecular Parasitology Laboratory, BRIC-National Institute of Animal Biotechnology, Hyderabad, Telangana, India; 2 Graduate Studies, BRIC-Regional Centre for Biotechnology, Faridabad, Haryana, India

**Keywords:** apicomplexa, *Toxoplasma gondii*, bromodomain protein, BDP4, BET inhibitors: I-BRD9

## Abstract

*Toxoplasma gondii*, an apicomplexan parasite, relies heavily on epigenetic regulation of gene expression, which is controlled by chromatin-modifying enzymes and histone acetylation, to invade and establish infection in the host. Bromodomain proteins are important epigenetic regulators in parasites, acting as “readers” of histone lysine acetylation to control gene expression. While many bromodomain proteins are unique to parasites, only a few have been explored for therapeutic applications. In this study, we characterized the parasite-specific bromodomain protein TgBDP4 as a key epigenetic reader and a potential drug target for toxoplasmosis. Protein-peptide interaction and pull-down studies reveal that TgBDP4 interacts strongly with acetylated histone H3 as well as unphosphorylated and Ser5-phosphorylated forms of RNA polymerase II-CTD, which are necessary for gene activation. Using a conditional knockdown approach, we demonstrate that TgBDP4 is essential for parasite survival in both cell culture and a mouse host. When the therapeutic potential of TgBDP4 was evaluated using three bromodomain inhibitors, I-BRD9, (+)-JQ1, and I-BET151, we found that I-BRD9, a selective inhibitor of HsBRD9, effectively inhibits TgBDP4 activity at much lower concentrations than HsBRD9. This inhibition occurs through the binding of I-BRD9 to conserved key residues of the acetyl-lysine-binding pocket of TgBDP4, resulting in a complete arrest of parasite replication in culture and extending the survival time of infected mice. Overall, this study highlights the indispensable role of TgBDP4-mediated gene regulation for parasite survival, establishing TgBDP4 as a promising drug target for treating toxoplasmosis.

*Toxoplasma gondii* is an intracellular protozoan parasite that belongs to the phylum Apicomplexa, which includes several significant pathogens affecting humans (*e.g.*, *Plasmodium*, *Cryptosporidium*) and animals (*e.g.*, *Neospora*, *Theileria*) (1). Infections caused by these parasites are difficult to treat or prevent, as they share eukaryotic cellular functions and have complex life cycles (2). *T*. *gondii* is a widely prevalent parasite that causes toxoplasmosis, a disease with serious health risks for developing fetuses, potentially leading to blindness and brain damage, as well as for immunocompromised individuals, potentially resulting in serious illness or death (2). The life cycle of *Toxoplasma* includes three infectious stages: tachyzoites, bradyzoites, and sporozoites. Tachyzoites multiply rapidly, bradyzoites grow slowly within tissue cysts, and sporozoites are found in oocysts shed in the feces of cats (3). The ability of *Toxoplasma* to switch between tachyzoites and bradyzoites is crucial for its pathogenesis and for maintaining a persistent infection (4). Current treatment involves a combination of pyrimethamine and sulfadiazine, which inhibits the rapidly dividing tachyzoite stage but has minimal activity against bradyzoites within tissue cysts, leaving chronic infection untreated (5). This treatment can also be toxic and cause significant adverse effects, underscoring the urgent need to identify new drug targets to develop safer, more effective treatments for toxoplasmosis.

Chromatin-based regulation in *Toxoplasma* is critical for coordinating the major transcriptional changes during developmental stage transitions (6, 7, 8). Histone lysine acetylation plays an important role in activating these transcriptional changes (8), as evidenced by studies showing that disrupting lysine acetyltransferases (9, 10) or lysine deacetylases (11, 12, 13) alters gene expression, inhibiting parasite proliferation and life cycle progression. An acetyl mark on a histone serves as a docking site for transcriptional regulators, which are recruited *via* their acetyl-lysine "reader" domain, the bromodomain (BRD) (14). The Bromodomains are ∼110-amino-acid sequences that feature a left-handed four-α-helix bundle structure, connected by two loops (ZA and BC), which form a hydrophobic pocket that recognizes and binds the acetyl group of a lysine residue (14). Variations in the sequence and length of the loops (ZA and BC) among different bromodomains likely contribute to their ability to recognize specific targets. After binding to their targets, bromodomain proteins recruit a variety of protein complexes to modify chromatin structure and regulate gene transcription. Due to the critical role of bromodomain proteins in transcriptional regulation, they are considered prime therapeutic targets for diseases such as cancer, inflammation, and metabolic disorders (15).

Bromodomain proteins have been found in protozoan parasites; however, in apicomplexans, they have been identified in only two species: *Plasmodium* and *Toxoplasma*. *Plasmodium* is predicted to have seven BRD proteins without direct orthologues outside the apicomplexan group (16), while *Toxoplasma* has twelve BRD proteins; six of these contain conserved bromodomains, whereas the other six do not have homologues in mammals, plants, or fungi (16). In *Plasmodium*, four BRD proteins have been characterized: GCN5 (17, 18), BDP1 (19, 20), BDP2 (21), and BDP7 (21). PfGCN5 is essential for the survival and transmission of the parasite, and its BRD domain is necessary for the acetylation of the variant histone H2B.Z at the promoters of euchromatic genes (17, 18). A complex composed of PfBDP1, PfBDP2, and PfBDP7 both supports the expression of invasion genes and functions as a repressor complex to ensure the mutually exclusive expression of variant surface antigens (21). Similarly, in *Toxoplasma*, four BRD proteins have been characterized: GCN5a (9), GCN5b (10), BDP1 (22), and BDP5 (23). TgGCN5a and TgGCN5b are two distinct lysine acetyltransferases with different preferences for histone H3 lysine acetylation sites and non-redundant functions; GCN5a is crucial for stress response (9, 24), and GCN5b is essential for parasite survival (10). Both parasite-specific BRD proteins, TgBDP1 (22) and TgBDP5 (23), are essential for tachyzoite growth, and their depletion leads to significant dysregulation of gene expression. While many identified BRD proteins can bind to acetylated histones and are deemed essential based on genome-wide CRISPR screens (25), only a few have been explored for their regulatory roles in gene expression or as potential drug targets in *Toxoplasma*.

Research on the inhibition of bromodomain and extraterminal (BET) proteins by small-molecule inhibitors is limited in parasites, with most studies focusing on the kinetoplastids (*Trypanosoma* and *Leishmania*). The effects of different human bromodomain inhibitors on *Trypanosoma cruzi* (26, 27, 28, 29, 30, 31) and *T*. *brucei* (32) were evaluated by examining their interactions with bromodomains and their impact on parasite replication. In *T*. *cruzi*, recombinant BDF3 (26) was observed to interact with both JQ1 and I-BET151, while BDF2 (29) exhibits specific binding to I-BET151. Similarly, in *T*. *brucei*, recombinant BDF2 (32) binds to I-BET151 and GSK2801, whereas BDF3 also interacts with I-BET151. All these compounds were effective in disrupting the growth of the parasite and altering its life cycle. Furthermore, recombinant LdBDF5 (33) binds to SGC-CBP30 and inhibits the growth of *Leishmania* promastigotes. In *Plasmodium falciparum*, the bromodomain inhibitors SGC-CBP30 (34) and MPM6 (35) demonstrated antiparasitic activity and were later shown to bind PfBDP1. The bromodomain inhibitor L-Moses (36) specifically targets the essential protein GCN5b in *T*. *gondii*, whereas I-BET-151 (37) has been reported to inhibit tachyzoite proliferation.

Due to the limited data on BET inhibitors and their targets in *Toxoplasma*, we characterized the parasite-specific bromodomain protein TgBDP4 as a potential drug target in this study. We show that TgBDP4 interacts strongly with acetylated histone H3, as well as unphosphorylated and Ser5-phosphorylated forms of RNAPII-CTD, which are crucial for gene activation. Using a conditional knockdown approach, we established that TgBDP4 is essential for parasite survival in both cell culture and a mouse host. Furthermore, when we assessed the therapeutic potential of TgBDP4 using three bromodomain inhibitors, we found that I-BRD9, a selective inhibitor of HsBrd9, effectively inhibits TgBDP4 activity by binding to its acetyl-lysine-binding pocket, leading to complete arrest of parasite replication in culture and increased survival time for infected mice. Overall, these findings reveal the potential of TgBDP4 as a target for the development of new anti-*Toxoplasma* compounds.

## Results

### TgBDP4 is a bromodomain-containing protein conserved in protozoan parasite

**Figure 1:**
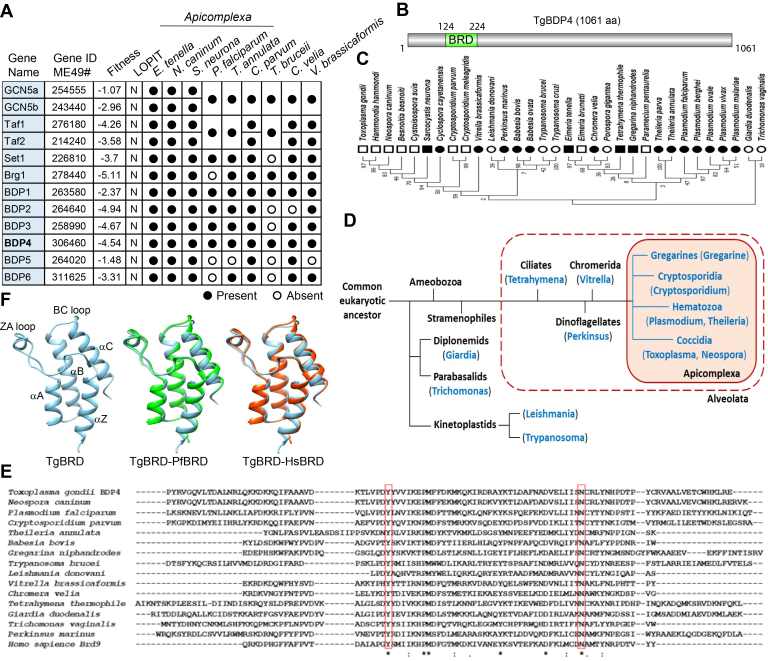
**Bromodomain-containing proteins, including BDP4 in apicomplexans.***A*, the conservation of bromodomain-containing proteins across protozoan parasites, including apicomplexans, was determined by a BLASTp search on VEuPathDB using protein sequences of *H*. *sapiens*. Summary of *T*. *gondii* bromodomain-containing proteins, indicating ToxoDB number, nuclear (N) localization based on hyperLOPIT, and fitness score derived from a genome-wide CRISPR fitness screen. *B*, schematic of TgBDP4 protein and domain architecture. *C*, a phylogenetic tree of bromodomain-containing proteins from protozoan parasites was constructed using MEGA analysis. This involved aligning full-length protein sequences with 100 bootstrap replicates. The nodes supported >50% are denoted. The open circle represents <500 aa, the solid circle 500 to 1000 aa, the empty square 1000 to 1500 aa, and the solid square >1500 aa. *D*, evolutionary tree with genera containing predicted TgBDP4 homologues in *blue*. Apicomplexans are in *yellow-shaded box*, and *red-dotted line* encompasses alveolates. Branch lengths are not to scale. *E*, multiple sequence alignment of bromodomain amino acid sequences from representative species, with TgBDP4 denoted as TGME49_306460 BDP4. The highly conserved tyrosine (Y) and asparagine (N) residues required for binding acetylated lysine are boxed in *red*. *F*, the predicted structure of TgBDP4 (124–224 aa) is shown in *sky blue*, generated using Swiss-model. The structure contains four α-helices connected by loops and forms a hydrophobic pocket for acetyl lysine binding. The predicted TgBDP4 structure (*sky blue*) is superimposed on *P*. *falciparum* BDP4 (*green*) and on human BRD9 (*orange*).

Bromodomain (BRD) proteins are present in most eukaryotic organisms, although their numbers can vary. In apicomplexans, BRD proteins have been identified in *Toxoplasma* and *Plasmodium* spp; however, their presence in other apicomplexan parasites remains unknown. To explore this, we performed BLASTp homology searches of apicomplexan genomes (38) (https://veupathdb.org/veupathdb/app/) using the amino acid sequences of *T*. *gondii* BRD proteins as queries. The *Toxoplasma* genome encodes 12 BRD proteins, all of which are essential for parasite survival, as indicated by a negative CRISPR fitness score derived from genome-wide CRISPR/Cas9 loss-of-function screens (25). All 12 BRD proteins are localized in the parasite nucleus based on the hyperLOPIT (Localization of Organelle Proteins by Isotope Tagging) data used to create a comprehensive subcellular atlas of the *T. gondii* proteome (39). *Neospora caninum*, *Eimeria tenella*, and *Sarcocystis neurona* also contain 12 BRD proteins each, while *Cryptosporidium parvum* have 11 (Fig. 1*A*). *Theileria annulata* contains 9 BRD proteins, whereas photosynthetic ancestors (*Chromera velia* and *Vitrella brassicaformis*) have 10 BRD proteins (Fig. 1*A*). Notably, *T*. *gondii*, *N*. *caninum*, *E*. *tenella*, and *S*. *neurona* all have two GCN5 homologues (GCN5a and GCN5b), whereas most other parasites, including apicomplexans and kinetoplastids, typically have only one (Fig. 1*A*).

Among the 12 BRD proteins identified in *Toxoplasma*, 6 (BDP1-BDP6) lack homologues in mammals, plants, or fungi (16). This study focuses on TgBDP4 (TGME49_306460), a predicted ∼110-kDa protein that contains a single bromodomain (Fig. 1*B*). TgBDP4 shows the highest overall sequence similarity to human BRD9 at 22% (Fig. S1) and 40% (Fig. S2) within the bromodomain among the BRD proteins, suggesting that TgBDP4 may be a BRD9-like protein in *Toxoplasma*. BLASTp homology searches identified homologues of BDP4 in all alveolate and kinetoplastid parasites (Fig. 1, *C* and *D*). The bromodomain of TgBDP4 contains the characteristic tyrosine (Y) and asparagine (N) residues essential for binding to acetylated lysines (Fig. 1*E*). The structure of the bromodomain of TgBDP4 was modeled using SWISS-MODEL (40). The predicted structure showed similarities to the bromodomain found in the human protein BRD9 and in *P*. *falciparum* BDP4 (Fig. 1*F*). It features a bundle of four anti-parallel alpha helices (αZ, αA, αB, and αC) connected by two loops (ZA and BC) (Fig. 1*F*), forming a hydrophobic binding pocket where the residues needed for recognizing and binding to acetylated lysine are appropriately positioned. Overall, based on the conserved sequence and predicted structure of the bromodomain, TgBDP4 is likely an acetyl-lysine reader.

### TgBDP4 strongly binds to acetylated histone H3 and phosphorylated RNAPII at Ser5

**Figure 2:**
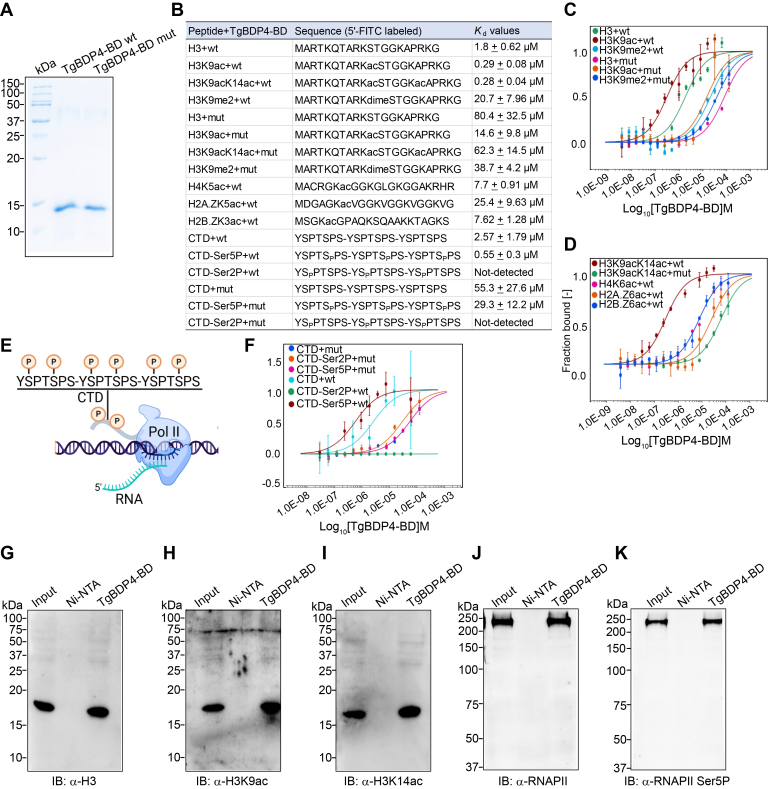
**Acetyl histone interaction activity of TgBDP4.***A*, purification profile of recombinant TgBDP4-BD wt and mut proteins analyzed on 15% SDS-PAGE. *B*, Peptide sequence of the FITC-labelled *T*. *gondii* histone variants (H3, H3K9ac, H3K9acK14Ac, H3K9me2, H4K5ac, H2A.ZK5ac, H2B.ZK3ac, CTD, CTD-Ser5P, and CTD-Ser2P) that were used for Microscale thermophoresis analysis (MST) are shown. *C* and *D*, interaction between TgBDP4 and the FITC-labelled Histone peptides was tested by Microscale thermophoresis analysis (MST), and the *K*_d_ values are indicated in (*B*). *E*, a schematic diagram illustrates the phosphorylated Ser2 and Ser5 residues located in the heptapeptide repeats (YSPTSPS) of RNA polymerase II. *F*, interaction of TgBDP4 with both the FITC-labeled unphosphorylated YSPTSPS and phosphorylated variants of YSPTSPS (YSpPTSPS and YSPTSpPS) was evaluated using MST, with *K*_d_ values mentioned in (*B*). *G*–*K*, pull-down followed by immunoblotting showing interaction of TgBDP4-BD with H3 (*G*), H3K9Ac (*H*), H3K14Ac (*I*), RNAPII (*J*), and RNAPII-Ser5P (*K*).

*Toxoplasma* BRD proteins are predicted to bind to acetyl-lysine residues on histone tails; however, their specific targets have not yet been identified. Here, we investigated the binding potential of the recombinant bromodomain (BD) of TgBDP4, TgBDP4-BD wt and TgBDP4-BD mut (replacing the conserved tyrosine 159 with phenylalanine and asparagine 197 with alanine, TgBDP4-BD mut - Y159F, N202A) proteins (Fig. 2*A*) to bind to acetylated histone H3, H4, H2A.Z, and H2B.Z peptides using microscale thermophoresis (MST). The *in vivo* acetylation status of these histones has already been established in *T*. *gondii* (41). To characterize the interactions between TgBDP4 and the histones, we designed and synthesized fluorescently labeled (FITC) 20-mer peptides (Fig. 2*B*) derived from the N-terminal regions (1–20 amino acids) of H3 (TgME49_261240), H4 (TgME49_239260), H2A.Z (TgME49_300200), and H2B.Z (TgME49_209910). These peptides included unmodified, monoacetylated (H3K9ac), diacetylated (H3K9acK14ac), and dimethylated (H3K9me2) variants of H3; monoacetylated H4 (H4K5ac); monoacetylated H2A.Z (H2A.ZK5ac); and monoacetylated H2B.Z (H2B.ZK3ac). In the MST-binding experiments, a fixed concentration of peptide (20 nM) was titrated against increasing concentrations of recombinant TgBDP4-BD wt or TgBDP4-BD mut protein, up to 125 μM (Fig. 2, *B*–*D*). Protein binding was measured as an increase in fluorescence, and the associated dissociation constants (*K*_d_ values) were calculated (Fig. 2*B*). The binding curves for TgBDP4-BD wt protein revealed that the H3K9acK14ac peptide exhibited the strongest binding (*K*_d_ = 0.28 ± 0.04 *μ*M), followed closely by H3K9ac (*K*_d_ = 0.29 ± 0.08 *μ*M) (Fig. 2, *B–D*), indicating specific interactions. Surprisingly, the unmodified H3 peptide showed weaker binding (*K*_d_ = 1.8 ± 0.62 *μ*M); however, this binding affinity was still higher than that of H4K5ac (*K*_d_ = 7.7 ± 0.91 *μ*M) and H2B.ZK3ac (*K*_d_ = 7.62 ± 1.28 *μ*M). The weakest binding was observed for H3K9me2 (*K*_d_ = 20.7 ± 7.96 *μ*M) and H2A.ZK5ac (*K*_d_ = 25.4 ± 9.63 *μ*M) (Fig. 2, *B*–*D*).

To investigate the significance of the conserved tyrosine 159 and asparagine 197 residues, the binding of the TgBDP4-mut protein to H3 variants was tested. The results showed that the TgBDP4-mut protein demonstrated very little binding to all the H3 variants (Fig. 2, *B*–*D*). These findings suggest that TgBDP4 has a strong association with acetylated H3 at K9 and K14 and that the conserved tyrosine 159 and asparagine 202 residues in the bromodomain are critical for this binding.

Mammalian BRD4 serves as a molecular link between activating histone acetylation marks and RNAPII transcription (41). The phosphorylation of the C-terminal domain (CTD) of RNAPII on the Y_1_S_2_P_3_T_4_S_5_P_6_S_7_ heptad repeats regulates transcription, with Serine 5 (Ser5) marking initiation and Serine 2 (Ser2) associated with the elongation (42) (Fig. 2*E*). Although these phosphorylated marks have also been observed in *Toxoplasma* (43, 44, 45, 46), the interaction between BRD proteins and RNAPII-CTD remains unexamined. To investigate this, we tested the ability of the recombinant TgBDP4-BD protein to bind with unphosphorylated, Ser5-phosphorylated, and Ser2-phosphorylated CTD peptides using MST (Fig. 2, *B* and *F*). To characterize the interactions between TgBDP4 and the CTD, we designed and synthesized fluorescently labeled (FITC) 21-mer peptides. These peptides contain three repeats of the YSPTSPS sequence in three different forms: unphosphorylated (CTD), Ser5-phosphorylated (CTD-Ser5P), and Ser2-phosphorylated (CTD-Ser2P). In the binding experiments, a 30 nM peptide was titrated against increasing concentrations of recombinant TgBDP4-BD wt or TgBDP4-BD mut protein, up to 125 μM. Protein binding was measured by fluorescence increase, and *K*_d_ values were calculated. The binding curves for TgBDP4-BD wt revealed that the CTD-Ser5P peptide showed the strongest binding (*K*_d_ = 0.55 + 0.3 *μ*M), followed by CTD (*K*_d_ = 2.57 + 1.79 *μ*M) (Fig. 2, *B* and *F*). No binding was observed for CTD-Ser2P. In contrast, TgBDP4-mut protein demonstrated very little binding to CTD (*K*_d_ = 55.3 + 27.6 *μ*M) and CTD-Ser5P (*K*_d_ = 29.3 + 12.2 *μ*M) (Fig. 2, *B* and *F*). Overall, interactions of TgBRD4 with acetylated H3, the CTD, and CTD-Ser5P indicate that TgBRD4 plays a crucial role in regulating transcription.

To confirm the binding of TgBDP4-BD to H3 and CTD peptides, we isolated nuclear extracts from RH tachyzoites. We then performed pull-down experiments using His6-tagged TgBDP4-BD and enriched the resulting complexes with Ni-NTA beads. Subsequently, we performed immunoblotting using anti-H3, acetylated H3, RNAPII, and RNAPII-Ser5P antibodies. Our findings indicate that the TgBDP4-BD protein strongly associates with H3 (Fig. 2*G*), H3K9ac (Fig. 2*H*), H3K14ac (Fig. 2*I*), RNAPII (Fig. 2*J*), and RNAPII-Ser5P (Fig. 2*K*). Together, these results are well supported by MST assays performed with the TgBDP4-BD and H3 and CTD peptides.

### TgBDP4 is essential for parasite replication

**Figure 3:**
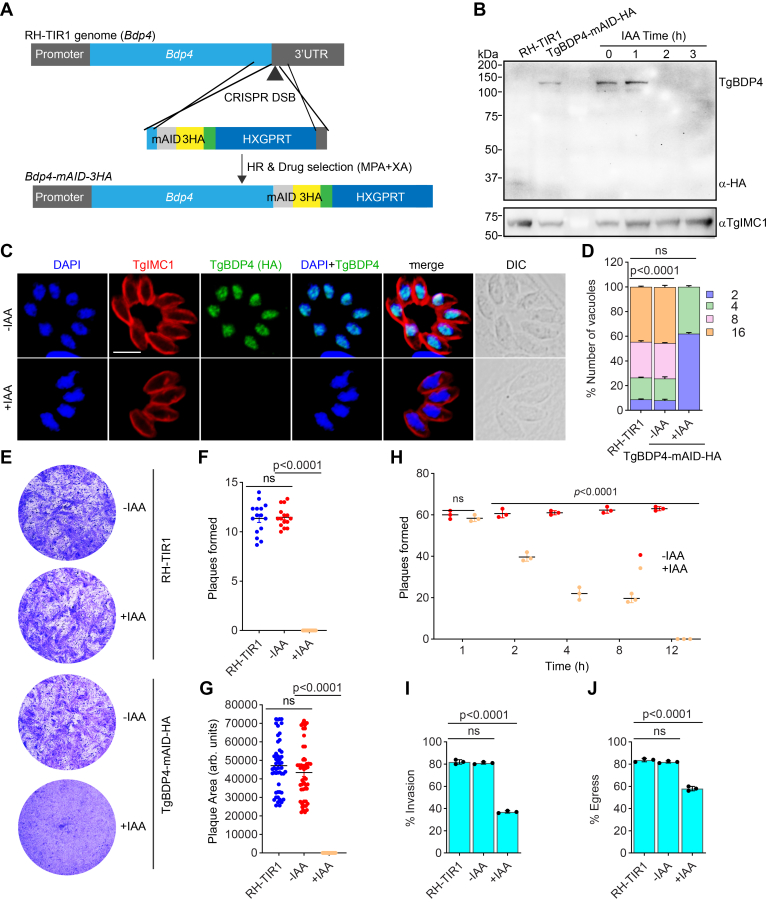
**Effect of TgBDP4 depletion on parasite replication.***A*, strategy for tagging of TgBDP4 protein in RH-TIR1-3FLAG parental line. *B*, Western blot analysis using anti-HA antibodies determined expression of TgBDP4-mAID-3HA in RH-TIR1-transfected parasites, with or without IAA, at the indicated time points. *C*, immunofluorescence analysis (IFA) of TgBDP4-mAID-HA grown with IAA or vehicle for 2 h (*n* = 3). Scale bar 5 μm. *D*, parasite replication. The graph shows the percentage of vacuoles containing the number of parasites of RH-TIR1 and TgBDP4-mAID-3HA parasites treated for 18 h with IAA or vehicle (*n* = 3) determined by IFA. *E*, plaque assay. Crystal violet-stained images of plaques formed by RH-TIR1 and TgBDP4-mAID-3HA parasites on HFF monolayer treated with IAA or vehicle (*n* = 3). *F* and *G*, quantification of plaque numbers (*F*) and plaque areas (*G*). *H*, quantification of plaques after IAA withdrawal at indicated time points in TgBDP4-mAID-3HA parasites. *I*, parasite invasion. The graph shows the percentage invasion of RH-TIR1 and TgBDP4-mAID-3HA parasites on HFF monolayer treated with IAA or vehicle, determined by IFA. *J*, parasite egress. The graph shows the percentage of egress of RH-TIR1 and TgBDP4-mAID-3HA parasites cultured with IAA or vehicle, followed by calcium ionophore treatment. IF analysis was performed to check intact or collapsed vacuoles. *D*–*J*; *n* = 3 replicates; mean ± S.D; one-way ANOVA; Dunnett’s comparison test; ns not significant; *p* values: ∗ < 0.05; ∗∗∗ < 0.001; ∗∗∗∗ < 0.0001.

TgBDP4 has a CRISPR score of −4.54 (25), indicating it is an essential gene; however, to provide experimental evidence, we utilized an auxin-inducible conditional knockdown approach (47, 48). We endogenously tagged TgBDP4 at the C-terminus with mini auxin-inducible degron (mAID) sequence fused with three copies of HA (3HA) (TgBDP4-mAID-3HA) in RH strain parasites that express TIR1 (Fig. 3*A*). This method allows rapid degradation of the TgBDP4-mAID-3HA protein upon addition of indole-3-acetic acid (IAA). Western blotting and immunofluorescence assays using an anti-HA antibody confirmed the expression of TgBDP4-mAID-HA in the parasites and its expected nuclear localization, as predicted by LOPIT (39). A complete loss of the TgBDP4-mAID-HA protein was observed 2 hours after the addition of IAA to the culture medium (Fig. 3*B*). Further immunofluorescence analysis also confirmed the absence of TgBDP4-mAID-HA protein, as no staining for TgBDP4-mAID-HA was observed in the IAA-treated parasites after 2 hours (Fig. 3*C*).

Next, we investigated how the depletion of TgBDP4 affects parasite-specific processes. The depletion of TgBDP4 resulted in a arrest of parasite replication. In the presence of IAA (+IAA), approximately 40% of vacuoles contained four parasites, while in the absence of IAA (-IAA), 50% contained 16 parasites (Fig. 3*D*). This indicates that parasite replication did not progress in the TgBDP4-depleted parasites. We also assessed the impact of TgBDP4 depletion on parasite viability using plaque assays. Unlike the parental strain and the -IAA condition, TgBDP4-depleted parasites produced no visible plaques (Fig. 3, *E*–*G*). To evaluate whether the parasites could recover from a temporary loss of TgBDP4 expression, we performed a plaque assay with six different IAA treatment regimens (Fig. 3*H*). We allowed the parasites to grow for 24 h, followed by treatment with either IAA or a vehicle control for 1, 2, 4, 8, or 12 h. After the treatment, the medium was replaced with IAA (or vehicle), and the cultures were incubated for an additional 5 days. The number of plaques formed was similar between the 1-h IAA and vehicle-treated parasites. In the 2-h IAA treatment, we observed a ∼33% reduction in plaque formation, whereas the 4- and 8-h IAA treatments showed ∼66% reductions (Fig. 3*H*). No plaques were observed after 12 h of IAA treatment, suggesting that there was no recovery of the parasites after 12 h of TgBDP4 depletion (Fig. 3*H*).

Furthermore, depletion of TgBDP4 significantly decreased the invasion efficiency of the parasites, with a ∼50% reduction in parasite invasion in IAA-treated parasites (Fig. 3*I*). Similarly, a ∼25% reduction in the ability of parasites to egress in response to calcium ionophore was observed following TgBDP4 depletion (Fig. 3*J*). Together, these data provide strong evidence that TgBDP4 is essential for the viability and proliferation of *Toxoplasma*.

### Depletion of TgBDP4 protects mice from lethal toxoplasmosis

**Figure 4:**
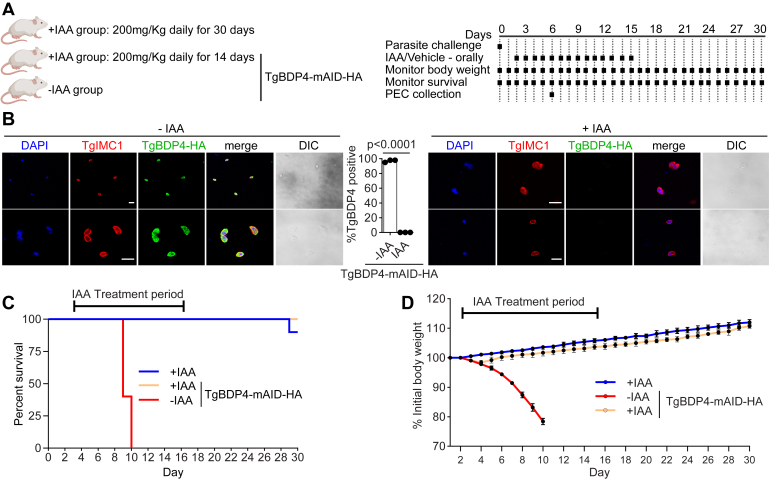
**TgBDP4 is essential for parasite survival in the mouse host.***A*, experimental design for *in vivo* examination of TgBDP4 essentiality (*n* = 10 BALB/c mice per group). *B*, mice were injected intraperitoneally (i.p.) with 100 TgBDP4-mAID-HA tachyzoites and treated with IAA or vehicle from days 2 to 15. On day 6, peritoneal exudate cells (PECs) were collected from one mouse in each of the -IAA and +IAA groups for IFA. The fixed cells were probed for parasites (rabbit α-TgIMC1/α-rabbit IgG Alexa Fluor 594) and TgBDP4-mAID-3HA (mouse α-TgHA/α-mouse IgG Alexa Fluor 488). The percentage of parasites expressing the TgBDP4 protein (±HA) was calculated. *n* = 3 replicates; mean ± SD; one-way ANOVA; Tukey’s posthoc test; *p* value: ∗ < 0.05; ∗∗∗ < 0.001; ∗∗∗∗ < 0.0001. Scale bar, 5 μm. *C*, a survival curve of the mice, including groups treated with IAA, those without IAA (infected with TgBDP4-mAID-HA), and those with IAA (infected with TgBDP4-mAID-HA), was plotted using the Gehan-Breslow-Wilcoxon test to compare differences, *p* < 0.0001. *D*, the mean body weight ± SD of the mice in the respective groups is presented.

The TgBDP4 protein is essential for the fitness of the parasite in tissue culture. To evaluate its significance in establishing infection within the host, we conducted mouse infection studies. We performed a 30-days survival experiment involving three groups of mice (10 per group): two groups were infected, and one group served as a control without infection (Fig. 4*A*). The mice in the two infection groups were injected intraperitoneally (i.p.) with 100 tachyzoites of TgBDP4-mAID-HA. From days 2 to 15 post-infection, these mice received oral treatment with either IAA or a vehicle (Fig. 4*A*). The third group, which was not infected, also received IAA. On day 6 post-infection, we sacrificed two mice from each infection group (both -IAA and +IAA) and collected peritoneal exudate cells (PECs) for analysis by immunofluorescence (Fig. 4*A*). In the -IAA infection group, all parasites expressed TgBDP4-mAID-HA as expected, while in the +IAA infection group, no parasites exhibited TgBDP4-mAID-HA, as confirmed by HA staining, which indicated successful depletion of TgBDP4 *in vivo* (Fig. 4*B*). TgIMC1 was used as a control protein, and its expression remained unchanged in both groups (Fig. 4*B*). By day 10 post-infection, all mice in the -IAA-infected group (receiving the vehicle) died from lethal toxoplasmosis, whereas IAA treatment rescued all the infected mice from fatal infection (Fig. 4*C*). In the +IAA non-infected group, no mortality was observed until the 29th day, with only one mouse dying on the 30th day. The -IAA-infected group showed severe morbidity and complete mortality compared to the +IAA-infected group. By day 3 post-infection, mice in the -IAA-infected group exhibited weight loss and signs of illness; however, no weight loss was observed in the +IAA-infected group (Fig. 4*D*). Depleting TgBDP4 expression effectively blocked *T*. *gondii* replication, as stopping IAA treatment after day 15 did not result in morbidity or mortality in the +IAA-infected group. We did not detect *T*. *gondii* DNA in peripheral blood mononuclear cells (PBMCs) or heart tissue from the mice in the +IAA infection group sacrificed on day 30 (data not shown). Overall, these results highlight the essential role of TgBDP4 in the survival and replication of the parasite within the mouse host.

### Determination of the therapeutic potential of TgBDP4

**Figure 5:**
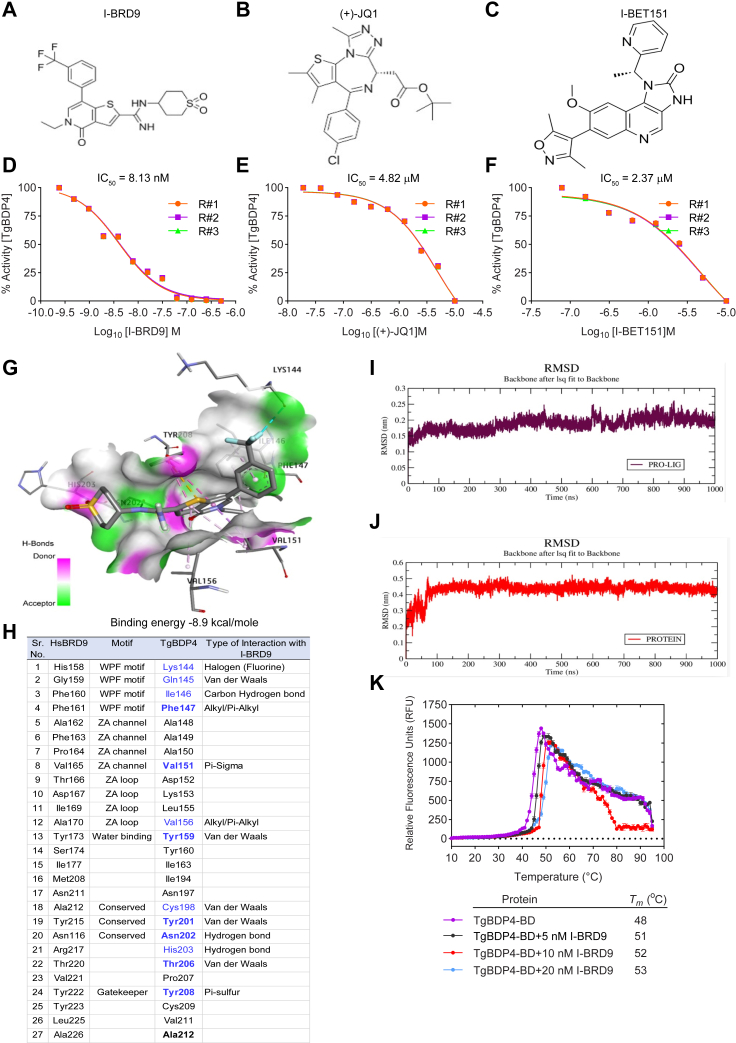
**Effect of I-BRD9, (+)-JQ1 and I-BET151 on TgBDP4 enzyme.***A*–*C*, chemical structure of I-BRD9 (*A*), (+)-JQ1 (*B*), and I-BET151 (*C*) compounds. *D*–*F*, determination of the median inhibitory concentration (IC_50_) of I-BRD9 (*D*), (+)-JQ1 (*E*), and I-BET151 (*F*) for TgBDP4-BD protein using a 10-dose response curve (*n* = 3). *G*, Proposed architecture of the TgBDP4-I-BRD9 complex. The structure was generated based on the modeled structure of TgBDP4. I-BRD9 was found to interact with TgBDP4 at the acetyl lysine binding site, with a free binding energy (Δ*G*_*bind*_) of −8.9 kcal/mol. *H*, the conservation of amino acids at the acetyl-lysine-binding site of HsBRD9 and TgBDP4 is shown. The amino acids that participate in bond formation in TgBDP4 are highlighted in blue. Identical amino acids in TgBDP4 that correspond to those in HsBRD9 are indicated in bold blue. *I* and *J*, the RMSD plot of the I-BRD9 complex with TgBDP4 (*I*) and alone (*J*) shows minimal deviation over 1000 ns, indicating stable interactions and structural stability. *K*. thermal stability assay showing the unfolding curve for the TgBDP4-BD wt- protein in the presence or absence of I-BRD9.

Given the essentiality of the TgBDP4 protein for parasite survival in cell culture and the mouse host, we evaluated the TgBDP4 as a potential therapeutic target. We selected three small-molecule compounds, I-BRD9, (+)-JQ1, and I-BET151 (Fig. 5, *A*–*C*), targeting bromodomain proteins with different specificities. I-BRD9 is a highly selective BRD9 inhibitor, while I-BET151 and (+)-JQ1 are pan-BET inhibitors. To determine if TgBDP4 is the molecular target of I-BRD9, (+)-JQ1, or I-BET151, we assessed the binding affinity of TgBDP4 with the H3K9ac peptide in the presence of these compounds using the MST assay (Fig. 5, *D*–*F*). We measured the half-maximal inhibitory concentration (IC_50_) of I-BRD9, (+)-JQ1, and I-BET151 in relation to TgBDP4 by analyzing the loss of binding with the H3K9ac peptide. All three compounds demonstrated concentration-dependent inhibition, with IC_50_ values of 8.13 nM for I-BRD9 (Fig. 5*D*), 4.82 μM for (+)-JQ1 (Fig. 5*E*), and 2.37 μM for I-BET151 (Fig. 5*F*). These results indicate that TgBDP4 activity was effectively inhibited by I-BRD9.

The human BRD9 bromodomain complexed with I-BRD9 shows that I-BRD9 binds to the acetyl-lysine binding pocket of human BRD9, facilitated by 25 specific residues within the binding site (49). To investigate whether I-BRD9 also binds to the acetyl lysine-binding pocket of the TgBDP4 and to identify the residues involved, we performed molecular docking using PyRx. We found that 11 of 27 amino acids in the acetyl-lysine-binding sites of HsBRD9 are conserved in the bromodomain of TgBDP4 (Fig. 5, *G* and *H*), suggesting that I-BRD9 similarly interacts with TgBDP4. These 11 conserved residues include the WPF motif, the ZA channel, the water-binding tyrosine, the conserved tyrosine and asparagine, and the gatekeeper tyrosine (Fig. 5*H*). The interaction model reveals that I-BRD9 binds to the acetyl-lysine-binding pocket of the TgBDP4 bromodomain, similarly, to how HsBRD9 binds. I-BRD9 forms a conventional hydrogen bond with Asn202 and His203, while also establishing van der Waals interactions with Tyr159, Gln145, Cys198, Tyr201, and Thr206. The residues Asn202 and Tyr159, which are conserved, play an important role in the interaction with acetyl lysine (Fig. 5*H*). Additionally, I-BRD9 forms a Pi-Sulfur bond with the gatekeeper residue Tyr208, a Pi-alkyl bond with Phe147 and Val156, a carbon-hydrogen bond with Ile146, and a halogen (fluorine) bond with Lys144 (Fig. 5, *G* and *H*). These interactions are crucial for acetyl-lysine binding within the TgBDP4 bromodomain. The significant changes in the binding regions modify their architecture, which may, in turn, affect the conformation of other residues in the ZA channel. The binding affinity of I-BRD9 for TgBDP4 is measured at −8.9 kcal/mol, indicating a strong interaction.

Subsequently, we performed a 1000-nanosecond simulation in GROMACS 2022.2 to assess the stable binding of I-BRD9 to TgBDP4 and the structural stability of the complex. The RMSD (Root Mean Square Deviation) plot clearly showed that the TgBDP4 and I-BRD9 complex (represented by the purple trajectory) (Fig. 5*I*) exhibits greater structural stability than TgBDP4 alone (represented by the red trajectory) (Fig. 5*J*). Throughout the 1000 ns simulation, the purple trajectory maintains a consistently low RMSD value of around 0.2 nm, indicating minimal deviation from the initial structure and suggesting a stable protein-ligand interaction (Fig. 5*I*). The lower RMSD values for the I-BRD9 and TgBDP4 complex indicate a more rigid and stable binding mode, supporting its potential as a promising candidate for targeting TgBDP4 and demonstrating enhanced structural integrity throughout the simulation.

To experimentally validate the *in silico* modeling results of I-BRD9 binding to TgBDP4, we performed a thermal shift assay to determine the melting temperature (*T*_*m*_) of the TgBDP4-BD in both the presence and absence of I-BRD9. Our results showed that as the concentration of I-BRD9 increased, the *T*_*m*_ values for TgBDP4-BD were significantly higher: 51 °C at 5 nM, 52 °C at 10 nM, and 53 °C at 20 nM compared to protein alone, which was 48 °C (Fig. 2*K*). This higher stabilization profile of TgBDP4-BD in the presence of I-BRD9 confirms their interaction and specificity. Overall, these Tm values are in good agreement with the binding affinities of TgBDP4-BD for acetylated H3, as observed in the MST and pull-down experiments.

### I-BRD9 inhibits tachyzoite proliferation and bradyzoite development

**Figure 6:**
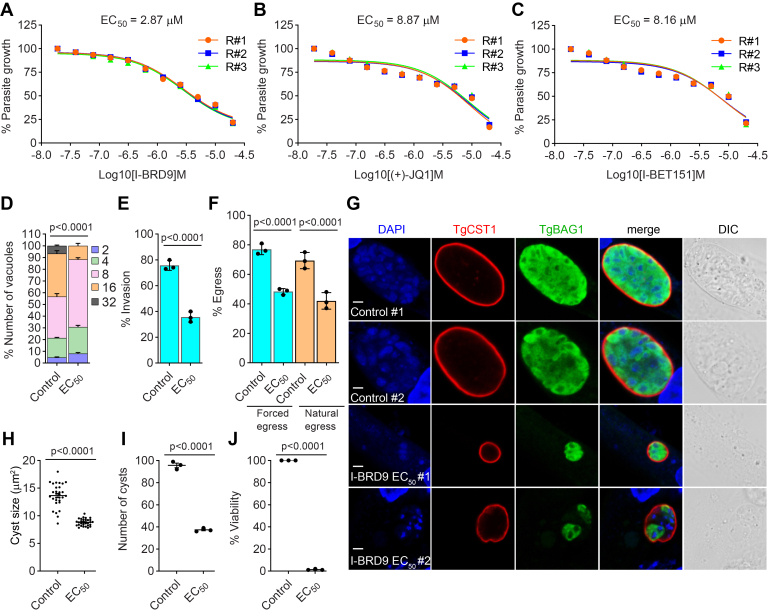
**Effect of I-BRD9, (+)-JQ1 and I-BET151 on parasite replication.***A*–*C*, determination of the half-maximal effective concentration (EC_50_) of I-BRD9 (*A*), (+)-JQ1 (*B*), and I-BET151 (*C*) against RH tachyzoites (*n* = 3). *D*, parasite replication. The graph shows the percentage of vacuoles containing the number of parasites treated for 36 h with I-BRD9 or vehicle determined by IFA (*n* = 3). *E*, parasite invasion. The graph shows the percentage of parasite invasion in an HFF monolayer treated with I-BRD9 or vehicle, determined by IFA (*n* = 3). *F*, parasite egress. The graph shows the percentage of parasites cultured for 48 h with I-BRD9 or vehicle, followed by either natural egress or calcium ionophore treatment to force egress. IFA was conducted to assess intact or collapsed vacuoles. *G*, IFA showing the effect of I-BRD9 on the development of *in vitro*-induced bradyzoite cysts (*n* = 3). Two representative IFA images are shown for control and I-BRD9 treatment. Scale bar 5 μm. *H*, cyst size measurement. The graph shows the size of the individual bradyzoite cysts after treatment with I-BRD9 or vehicle. *I*, cyst number measurement. The graph shows the number of bradyzoite cysts after treatment with I-BRD9 or vehicle. *J*, Bradyzoite viability after treatment with I-BRD9 or vehicle as assessed by plaque assay. *D*–*F*, *H*, and *I*, *n* = 3 replicates; mean ± SD; one-way ANOVA; Dunnett’s comparison test; ns not significant; *p* values: ∗ < 0.05; ∗∗∗ < 0.001; ∗∗∗∗ < 0.0001.

Given that all three compounds, I-BRD9, (+)-JQ1, and I-BET151, inhibited the TgBDP4 activity in interaction with the H3 peptide, we subsequently tested their effects on tachyzoite growth using a plaque assay (Fig. 6, *A*–*C*). Each compound was evaluated over a concentration range of 0.02 μM to 20 μM. I-BRD9 demonstrated the ability to inhibit the growth of RH parasites, with an effective concentration (EC_50_) of 2.87 μM (Fig. 6*A*). In contrast, I-BET151 and (+)-JQ1 showed an effective concentration (EC_50_) of 8.16 μM and 8.87 μM, respectively (Fig. 6, *B* and *C*). These results suggest that TgBDP4 may be a BRD9 homolog in *T*. *gondii*, as it can be effectively inhibited both *in vitro* and *in vivo* by I-BRD9.

The depletion of TgBDP4 led to an arrest in replication, severely impairing the parasite's ability to invade and exit host cells. Based on this finding, we investigated the effects of inhibiting TgBDP4 with I-BRD9 on these processes using EC_50_ concentration of the compound. EC_50_ concentration of I-BRD9 significantly inhibited parasite replication, as demonstrated by the absence of vacuoles containing 32 parasites, a lower percentage of vacuoles containing 16 parasites, and a notable increase in vacuoles with eight parasites (Fig. 6*D*). In contrast, as expected, the control parasites displayed vacuoles with 32 parasites, a higher percentage of vacuoles containing 16 parasites. Furthermore, treatment with EC_50_ concentration of I-BRD9 significantly reduced the parasite's ability to invade host cells (Fig. 6*E*). The efficiency of parasite egress was also notably diminished following I-BRD9 treatment, both under natural egress conditions and during forced egress induced by calcium ionophores (Fig. 6*F*).

Further, we evaluated the impact of I-BRD9 on bradyzoite replication by measuring both the size and number of bradyzoite cysts. We found both the EC_50_ concentration of I-BRD9 significantly reduced cyst size (Fig. 6, *G* and *H*), an indicator of bradyzoite growth, as well as the number of cysts (Fig. 6*I*). Next, to determine the viability of the bradyzoites within these cysts, we disrupted the cysts, used trypsin to release the bradyzoites, and cultured them on HFF monolayers to assess plaque formation. The bradyzoites treated with the EC_50_ concentration of I-BRD9 showed near-zero viability, as evidenced by only 1 to 2% plaques formed compared to those without I-BRD9 treatment (Fig. 2*J*).

### I-BRD9 treatment offers partial protection in the infected mice

**Figure 7:**
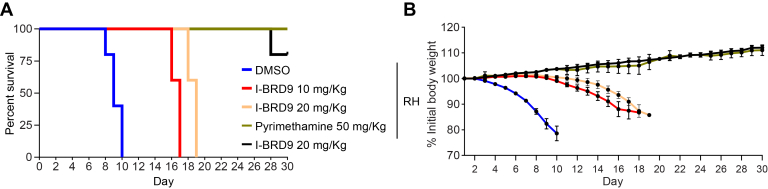
**Effect of I-BRD9 on the growth of the parasite in the infected mouse.***A*, effect of I-BRD9 on lethal toxoplasmosis was tested using BALB/c mice infected with the RH strain. Survival curve of mice from five groups. 1. DMSO with infection (100 RH tachyzoites). 2. I-BRD9 10 mg/kg with infection (100 RH tachyzoites), 3. I-BRD9 20 mg/kg with infection (100 RH tachyzoites), 4. Pyrimethamine 50 mg/kg with infection (100 RH tachyzoites), 5. I-BRD9 20 mg/kg no infection. The Gehan-Breslow-Wilcoxon test was used to compare survival curves. Ns not significant. Mortality due to acute toxoplasmosis was observed in the DMSO with infection group and the I-BRD9 10 mg/kg with infection group, as confirmed by IFA (*n* = 3). *B*, the mean body weight ± SD of the mice in the respective groups.

After demonstrating that I-BRD9 is effective against both asexual stages of *T*. *gondii*, we further investigated its potential protective effect against lethal toxoplasmosis using a mouse infection model. To evaluate the efficacy of I-BRD9 *in vivo*, mice were infected with 100 RH tachyzoites and subsequently treated with either 10 mg/kg or 20 mg/kg of I-BRD9 for 10 days post-infection (pi). Previous studies in mice indicated that the 20 mg/kg dose is well-tolerated and does not cause toxicity. For the positive control, one group of mice received pyrimethamine (50 mg/kg) for 10 days following the infection. As expected, all mice died by day 10 pi in the control group, and no mortality was observed in the pyrimethamine-treated mice (Fig. 7*A*). I-BRD9 treatment extended survival in mice (Fig. 7*A*), with less weight loss than in control (Fig. 7*B*); however, it did not protect infected mice in either dosage group, as all mice succumbed by day 20 pi (17 days in the 10 mg/kg group and 19 days in the 20 mg/kg group). In uninfected mice receiving 20 mg/kg of I-BRD9, only one mouse died on day 28, indicating no I-BRD9-induced toxicity. These findings suggest that I-BRD9 could be a promising candidate for further development, though it requires optimization for *in vivo* applications.

## Discussion

Bromodomain proteins are important epigenetic regulators in protozoan parasites, acting as 'readers' of histone lysine acetylation to control key gene expression, making them promising new drug targets. In this study, we characterized the *Toxoplasma* bromodomain protein TgBDP4 as a potential drug target. TgBDP4 is found in apicomplexan and kinetoplastid parasites and is highly similar to human BRD9. TgBDP4 interacts strongly with acetylated histone H3 and phosphorylated RNAPII-CTD at Ser5, both of which are important for gene activation. Using a conditional knockdown approach, we demonstrate that TgBDP4 is essential for the parasite's survival in both cell culture and a mouse host. When its therapeutic potential was evaluated using three bromodomain inhibitors, we found that I-BRD9, a selective inhibitor of HsBRD9, effectively inhibits TgBDP4 activity at much lower concentrations than HsBRD9 by binding to its acetyl-lysine-binding pocket. Consequently, I-BRD9 leads to a complete halt in parasite replication in culture and increases the survival time of infected mice.

Bromodomain proteins are recognized as significant epigenetic regulators in protozoan parasites like *Plasmodium*, *Trypanosoma*, *Leishmania*, and *Toxoplasma* (16). Most of these proteins are essential for parasite survival and play crucial roles in regulating the expression of key genes required for processes such as host cell invasion, stage differentiation, virulence, antigenic variation, and responses to stress. To identify the target genes of bromodomain proteins, researchers have employed a combination of ChIP-seq to map the binding sites of these proteins in the genome and RNA-seq in bromodomain mutants to determine which genes are transcriptionally affected, with the overlap indicating the direct targets (17, 18, 19, 21, 22). Our investigation into peptide-protein interaction and pull-down studies indicates that the acetyl-lysine reader protein TgBDP4 regulates global RNAPII transcription. Depletion of TgBDP4 results in a complete halt of parasite replication, significantly reducing both parasite invasion and egress. The pronounced impact of TgBDP4 depletion on various parasite processes suggests that TgBDP4 may not only regulate the expression of specific genes but could also influence a broader range of genes. While identifying the target genes and their regulation by TgBDP4 is beyond the scope of this study, our findings support the potential of TgBDP4 as a target for developing new drugs to combat toxoplasmosis.

In protein–peptide interactions experiments, we directly measured the binding affinities of the TgBDP4 bromodomain for peptide ligands derived from histones H3, H4, H2A.Z, and H2B.Z that carry an acetylation PTM. Our results from the MST analysis demonstrated that mono-acetylated H3 (H3K9ac) and di-acetylated H3 (H3K9acK14ac) are the preferred ligands of the TgBDP4 bromodomain, exhibiting the highest affinities compared to mono-acetylated histones H4, H2A.Z, and H2B.Z PTMs. This protein–peptide interaction was also confirmed by pull-down assay, suggesting that the H3K9ac modification likely drives the interaction of the TgBDP4 bromodomain with acetylated histones. Histone modifications H3K9ac and H3K14ac, particularly in combination, are generally associated with euchromatin and are found abundantly at the transcription start sites (TSS) of actively transcribed genes and enhancers in model eukaryotes (50). Similarly, in *Plasmodium* (17) and *Toxoplasma* (51), the essential histone acetyltransferase GCN5 targets H3 at lysines K9 and K14, playing a crucial role in regulating the expression of numerous genes vital for parasite replication and viability. Thus, our MST data demonstrating a strong affinity of the TgBDP4 bromodomain for the histone H3K9acK14ac ligand further support a role for TgBDP4 in gene activation *via* its interaction with di-acetylated H3 histones.

In eukaryotes, productive transcription depends on the phosphorylation of the CTD of RNAPII (42). The CTD consists of repeated heptad sequences of Y_1_S_2_P_3_T_4_S_5_P_6_S_7_, which vary in number among different organisms. Phosphorylation of the CTD residues serine 5 (Ser5) and serine 2 (Ser2) is essential for transcription initiation and elongation, respectively (42). In *T*. *gondii*, the RNAPII-CTD contains 10 heptad sequences, represented as YSPxSPx (where x can be any amino acid), that retain the conserved Ser5 and Ser2 (43, 44). As in mammalian cells, phosphorylation at Ser5 by TgCrk7 and at Ser2 by TgCrk9 plays vital roles in transcription initiation and elongation in *T*. *gondii* (43, 44, 45, 46). Our RNAPII-TgBDP4 interaction studies show that TgBDP4-BD binds with both the unphosphorylated CTD and the Ser5-phosphorylated CTD but not with the Ser2-phosphorylated CTD of TgRNAPII. This suggests that TgBDP4 may facilitate transcription initiation by recruiting transcription machinery to the promoter, thereby activating gene expression, as supported by its interaction with acetylated Histone H3. The direct interaction between TgBDP4-BD and CTD-Ser5P is a new finding. In mammals, BRD proteins, especially BRD4, are known to function as atypical kinase (where other CTD kinases are inactive) that binds to the CTD and specifically phosphorylate only Ser2 (52). Generally, BRD4 interacts with RNAPII and acts as a positive regulator of transcription, facilitating the transition from initiation to elongation (53). Therefore, it is intriguing to investigate the genome-wide distribution of TgBDP4 alongside RNAPII to confirm its role in transcription initiation in *Toxoplasma*.

The affinity of TgBDP4-BD for I-BRD9 (8.13 nM) was much lower than that of I-BET151 (2.37 μM) and (+)-JQ1 (4.82 μM). Both I-BET151 and (+)-JQ1 are among the most extensively studied pan-BET inhibitors, with varying IC_50_ values in the nanomolar (nM) range depending on the specific target bromodomain (54, 55). In contrast, I-BRD9 is a specific and potent inhibitor of BRD9 protein, demonstrating selectivity over 70-fold for BRD9 relative to other BRD proteins, with an IC_50_ of approximately 50 nM (49). I-BRD9 achieves this selectivity through unique interactions, such as hydrogen bonds with Asn100 and π-stacking, rather than merely blocking the acetylated lysine site that is common to all bromodomains (49). The strong affinity of TgBDP4-BD for I-BRD9 can be attributed to the relatively high homology between TgBDP4 and the human BRD9 protein, as well as the presence of half of the specific residues in the acetyl binding pocket necessary for the formation of hydrogen bond, van der Waals bond, and Pi-Sulfur bond during the interaction between BRD9 and I-BRD9. Results from *in silico* evaluation and thermal shift assay of the interactions between TgBDP4 and I-BRD9 align with the RMSD findings, indicating that I-BRD9 exhibits greater structural stability and holds promise as a candidate for targeting TgBDP4, which lacks a homologue in mammals, plants, or fungi.

The reduced parasite proliferation also highlights the inhibitory effect of I-BRD9 on the interaction between TgBDP4-BD and acetylated histone H3. I-BRD9 inhibits the rapid multiplication of tachyzoites and the growth of bradyzoite cysts at a significantly lower concentration (EC_50_ = 2.87 μM) than the cytotoxicity threshold observed in HEK293 cells (33 μM) (56). However, despite its target-specific inhibition *in vitro*, I-BRD9 was ineffective in treating lethal toxoplasmosis, except for extending survival in mice (the natural host) at a dose of 20 mg/kg. This ineffectiveness may be attributed to factors such as higher dose requirements, poor bioavailability, rapid efflux, or metabolic breakdown. To address these limitations, a newly developed PROTAC (Proteolysis-Targeting Chimera) variant of I-BRD9, currently in clinical trials and with improved oral bioavailability in mice, may prove beneficial.

**Figure 8:**
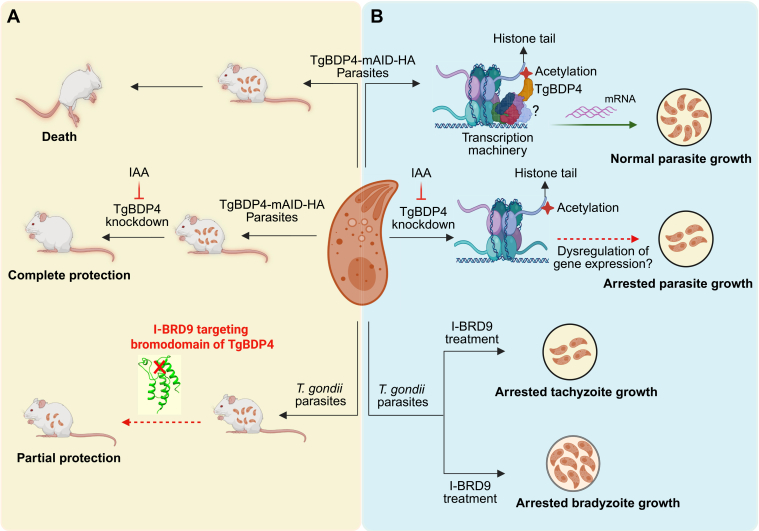
**An illustration showing the effects of TgBDP4 depletion on parasites.** TgBDP4 regulates gene expression, which is essential for normal parasite growth and replication (*A* and *B*). The absence of TgBDP4 resulted in parasite replication arrest (*B*) and rendered the parasites non-viable, ultimately preventing lethal toxoplasmosis (*A*). By leveraging insights from both *in vitro* and *in vivo* studies, the strategic application of a small-molecule inhibitor, I-BRD9 or its derivatives, specifically designed to target TgBDP4, offers a promising approach to block TgBDP4 effectively. This approach may replicate the observed effects *in vivo* and could provide a way to manage lethal *T. gondii* infection. The illustration was created using BioRender.

Overall, our work provides a detailed characterization of the parasite-specific bromodomain protein TgBDP4, an epigenetic reader of acetylated lysine histones in *Toxoplasma*. TgBDP4 is essential for parasite growth in culture and in a mouse host, and can be effectively inhibited by the compound I-BRD9. I-BRD9 treatment exerts an anti-proliferative effect on parasites and extends survival in infected mice. This compound serves as a valuable starting point for the development of more potent inhibitors of TgBDP4 for drug discovery aimed at combating *T*. *gondii* infection (Fig. 8).

## Experimental procedures

### Ethics statement

The laboratory research protocol (IBSC/April-2026/NIAB/AD018) and the animal research protocol (IAEC/NIAB/2024/09/ASD) have been approved by the ethics committee at the National Institute of Animal Biotechnology.

### Parasite culture

*T. gondii* strains RH (#50174, ATCC), RH-TIR1 (#NR-51145, BEI), RH TgBDP4-mAID-HA, and ME49 (#NR-20729, BEI) were maintained in primary human foreskin fibroblasts (HFF, #CRL-1634, ATCC) cells in Dulbecco's modified Eagle's medium supplemented with 10% FBS, 2mM L-glutamine, 10 μg/ml gentamicin, 1% penicillin/streptomycin, and at 37 °C and 5% CO_2_ (57). To purify the parasites for experiments, infected HFF cells were scraped, passed through a 27-gauge needle, and then filtered through a 3.0 μM filter to remove host cell debris.

### Gene cloning, expression and TgBDP4 protein purification

Sequence encoding bromodomain of TgBDP4 (TgBDP4-BD: 328–684 bp/109–228 aa) was PCR amplified (Table S1) from *T*. *gondii* cDNA and cloned into pET-28a vector (#69864, Sigma) between *Nde*I-*Xho*I sites. TgBDP4-BD recombinant protein with an N-terminal 6xHis-tag (∼15 kDa) was expressed in *E.* *coli* BL21 Rosetta (#70954, Novagen) and purified using Ni-NTA agarose (#30210, Qiagen), as previously described (58). Briefly, *E*. *coli* transformed with TgBDP4-BD was cultured overnight in 10 ml of Luria-Bertani (LB) medium containing 50 μg/ml kanamycin and 34 μg/ml chloramphenicol. This culture was transferred to 1000 ml of LB medium with the same antibiotics and incubated at 37 °C. Upon reaching an *OD*_600_ of 0.4, 1 mM IPTG was added, and incubation continued at 25 °C for 16 h. Cells were harvested by centrifugation, and the pellets were resuspended in 40 ml of lysis buffer (50 mM NaH_2_PO_4_, 300 mM NaCl, 100 μg/ml lysozyme, 10 mM imidazole, 0.1% Triton X-100, and 0.3 mM PMSF, pH 8.0]. The His-tagged protein was purified from the supernatant using Ni^2+^-NTA resin and eluted with 300 mM imidazole. The purified protein was dialyzed in 1× PBS and stored at −80 °C. The TgBDP4-BD mutant construct, which replaces the conserved tyrosine at position 159 with phenylalanine and the asparagine at position 202 with alanine (TgBDP4-BD mut 109–228 aa - Y159F, N202A), was synthesized in the pET-28a vector between *Nde*I-*Xho*I sites by Genescript. The recombinant TgBDP4-BD mut protein was purified using the same method as the wild-type protein.

### Microscale thermophoresis (MST) assay

MST assays were conducted using a Monolith NT.115 (NanoTemper) (59). In these assays, 20 nM of each FITC-labeled histone peptide (H3, H3K9ac, H3K9acK14ac, H3K9me2, H4K5ac, H2A.ZK5ac, and H2B.ZK3ac) was titrated against the TgBDP4-BD wt or TgBDP4-BD mut protein, which was serially diluted from 125 μM to 60 nM in MST buffer (50 mM Tris-HCl, 150 mM NaCl, 10 mM MgCl2, and 0.05% Tween 20). For the TgRNAPII-CTD experiments, 30 nM of each FITC-labeled RNAPII-CTD peptide, containing three repeats of the sequences YSPTSPS, YSpPTSPS, and YSPTSpPS, was similarly titrated against the TgBDP4-BD wt or TgBDP4-BD mut protein, diluted from 125 μM to 60 nM in the same MST buffer. The reactions were incubated at 25 °C for 15 min, then loaded into glass capillaries (NanoTemper). Fluorescence intensity was measured using 20% infrared laser power and 20% light-emitting diode intensity. The data were analyzed with Affinity Analysis software version 2.3 (NanoTemper) to determine the binding affinity (dissociation constant, *K*_d_) between TgBDP4-BD wt or TgBDP4-BD mut and various histone/RNAPII-CTD peptide variants.

### Ni-NTA pull-down assays and immunoblotting

Pull-down experiments were performed as previously described (60) using nuclear extract and recombinant TgBDP4-BD protein. Approximately 5 × 10^8^ RH tachyzoites were used to prepare nuclear extract (61) for each pull-down reaction. In each reaction, 50 μg of nuclear extract was mixed with 5 μg of recombinant TgBDP4-BD protein and incubated at 4 °C on a nutator for 60 min. Following this, 50 μl of Ni-NTA agarose was added, and the mixture was further incubated for another 60 min at 4 °C. The beads were washed five times using 1 ml of wash buffer, which consisted of 50 mM HEPES (pH 7.5), 500 mM KCl, 2 mM EDTA, and 0.1% NP-40. The bound proteins were then eluted by incubating the beads in 20 μl of elution buffer, containing 50 mM HEPES (pH 7.5) and 500 mM imidazole, at 4 °C on a nutator. Eluted samples were separated on a 15% (for Histones) or 10% (for RNAPII) SDS-PAGE gel and transferred onto a 0.45 μm PVDF membrane as described in the immunoblotting method. Immunoblotting was performed overnight at 4 °C with the following primary antibodies (1:1000 dilution in PBS): H3 (#9715S, CST), H3K9ac (#MA5-33384, Invitrogen), H3K14ac (#07-353, Sigma), RNAPII (#1862243, Invitrogen), and RNAPII-Ser5P (#ab5131, Abcam). The membranes were washed with PBST buffer thrice, and blots were then incubated with the HRP-conjugated α-mouse or α-rabbit secondary antibodies (1:5000 dilution in PBS). The membranes were rewashed three times with PBST buffer, and protein bands were visualized *via* chemiluminescence using SuperSignal West Femto substrate.

### Generation of auxin-inducible TgBDP4-mAID-3HA transgenic parasites

TgBDP4-mAID-3HA parasites were generated by CRISPR/Cas9-mediated site-specific gene editing using the RH TIR1-3FLAG strain of *T*. *gondii* (47, 48). A CRISPR/Cas9 plasmid was generated with a specific guide RNA targeting the 3′ end of Tg*Bdp4* (Table S1). This was accomplished by mutating the UPRT sequence from the pSAG1::Cas9-U6::sgUPRT plasmid (#54467, Addgene) using a Q5 Hot Start site-directed mutagenesis kit (#E0554S, NEB) and with specific primers (Table S1). The process aimed to induce double-strand DNA breaks and facilitate the insertion of the PCR fragment. The PCR fragment containing the mAID-3HA tag and the HXGPRT selection was amplified from the plasmid pTUB1:YFP-mAID-3HA (#87259, Addgene) with 40 bp of homology with the 3′ end of BDP4 to facilitate insertion through double homologous recombination. RH TIR1-FLAG parasites were cotransfected with 15 μg each of TgBDP4-mAID- 3HA amplicon and pSAG1::Cas9-U6::sgBDP4 plasmid by electroporation using the Gene Pulser Xcell Total System (#1652660, BioRad). Transfected parasites were drug-selected with mycophenolic acid (25 μg/ml) and xanthine (50 μg/ml) for 3 growth cycles before cloning by serial dilution. Endogenous tagging of mAID-HA (TgBDP4-mAID-HA) was verified using immunoblotting and immunofluorescence (IF) staining. The auxin-induced degradation of TgBDP4-mAID-HA was tested by culturing the parasites in a medium containing 500 μM indole-3-acetic acid (IAA) (#I2886, Sigma), followed by immunoblotting and immunofluorescence (IF) staining with α-HA antibody (#H3663).

### Immunoblotting

Filter-purified parasites were suspended in SDS-PAGE sample buffer, boiled for 10 min, and loaded ∼20 μg/well of a 10% polyacrylamide gel. The gel was transferred to a 0.2 μm PVDF membrane (#1620177, BioRad) using a Trans-Blot System (#1703930, BioRad) at 100V for 2 h. The membrane was blocked in 5% non-fat milk in PBS for 60 min, then probed overnight at 4°C with a primary antibody [α-HA/TgIMC1 (46) −1:5000] in PBS. After five washes with PBST (PBS with 0.1% Tween-20), the membrane was probed with either HRP-conjugated α-mouse-IgG (#Sc-2005, Santa Cruz) or α-rabbit (#Sc-2537, Santa Cruz), rewashed, developed using the Clarity Western ECL kit (#1705056, BioRad), and visualised on a ChemiDoc Imager (#12003153, BioRad).

### Immunofluorescence (IF) staining

HFF monolayers were grown on coverslips and subsequently infected with tachyzoites of RH-TIR1 or RH TgBDP4-mAID-HA. The cells were fixed and permeabilized in PBS containing 2% paraformaldehyde and 0.05% Triton X-100. Following this, the cells were blocked with 5% BSA and incubated with primary antibodies (α-HA/TgIMC1 - 1:100 in PBS) at 25 °C for 1 h. After five washes, cells were incubated with secondary antibodies (Alexa Fluor-488 or 594-conjugated α-mouse IgG or α-rabbit IgG, 1:1000/PBS) at 25 °C for 1 h. Coverslips were mounted on a glass slide with Vectashield medium containing DAPI (#H-1200–10, Vector Labs). Images were captured with a Leica Confocal microscope with a 63 × objective and processed with LAS X software.

### Parasite replication assay

HFF monolayers on glass coverslips in a 6-well plate were inoculated with 10^3^ RH-TIR1 or RH TgBDP4-mAID-HA parasites per well and incubated for 12 h. Next, the medium was removed, and the parasites were cultured in the presence of 500 μM IAA or MeOH vehicle for 18 h. Subsequently, the cells were fixed and permeabilized in PBS containing 4% paraformaldehyde and 0.05% Triton X-100, followed by IFA using α-TgIMC1 (1:1000 in PBS) and DAPI to visualize individual parasites. The number of parasites in each vacuole was determined by counting parasites in 50 randomly selected vacuoles for each experiment (*n* = 3).

### Plaque assay

Freshly egressed 100 RH-TIR1 or RH TgBDP4-mAID-HA parasites were inoculated onto HFF monolayers in a 6-well plate per well. After 6 h, the parasite medium was replaced with medium containing 500 μM IAA or MeOH vehicle. After 5 days, cells were fixed with 100% ice-cold methanol for 20 min and stained with a 2% crystal violet solution for 20 min. Plaques observed as clear zones of lysis (white) against intact cells (purple). Plaque numbers were counted by examining 50 random fields for each experiment (*n* = 3). Plaque areas were quantified using ImageJ software. In the rescue experiment, the IAA-containing medium was replaced with standard parasite medium at specified time points, and plaque numbers were counted as described above.

### Invasion assay

Freshly egressed RH-TIR1 or RH TgBDP4-mAID-HA parasites were incubated with a medium containing IAA or MeOH at 37 °C for 60 min. Subsequently, 10^3^ parasites were allowed to infect HFFs grown on glass coverslips for 30 min at 37 °C, followed by fixation with 4% paraformaldehyde. A non-permeabilizing IFA was performed by staining extracellular parasites with rabbit α-TgSAG1 antibodies (1:2000 in PBS) (59), followed by permeabilization with PBS/0.05% Triton X-100 and staining with mouse α-TgIMC1 (1:2000 in PBS). Cells were stained with Alexa Fluor-conjugated secondary antibodies (anti-mouse IgG AF 488 and anti-rabbit IgG AF 594) and DAPI. Parasites (green/red) were counted in fifty random fields, and the relative efficiency of attachment and invasion by IAA-treated parasites was expressed as the mean percentage of the vehicle treatment across three independent experiments.

### Egress assay

Freshly egressed RH-TIR1 or RH TgBDP4-mAID-HA parasites were inoculated on HFF monolayers grown on glass coverslips. After 20 h, the medium was replaced with DMEM containing IAA or MeOH, and cells were further incubated for 8 h. To induce parasite egress, 3 μM calcium ionophore A23187 was added to the medium and incubated for 2 min. Cells were then fixed, permeabilized, and stained with antibodies α-TgGRA2 (59) (1:1000 in PBS) and α-TgIMC1 (1:2000 in PBS). Sixty vacuoles were examined to count intact and collapsed vacuoles for each (*n* = 3), with vacuoles containing fewer than two parasites considered intact.

### Mouse infection

The degradation of the RH TgBDP4-mAID-HA protein in mice, induced by auxin, was carried out as previously described (62, 63). A 30-days survival experiment was performed with 6-week-old male BALB/c mice, divided into three groups of 10. Two groups were injected intraperitoneally (i.p.) with 100 tachyzoites of TgBDP4-mAID-HA. On the second day post-infection (pi), one group received IAA, while the other was given a methanol vehicle with 5% sucrose in their drinking water. The third group served as a no-infection control and was also administered IAA. The IAA was administered for 14 days (2 days pi to 15 days pi) using drinking water (0.5 mg/ml) and oral gavage (12.5 mg/ml). All mice were weighed and monitored daily. To assess the depletion of the TgBDP4 protein, two mice were sacrificed by CO_2_ asphyxiation on day 6 pi. Peritoneal exudate cells (PECs) were collected and analyzed using IF staining with α-HA (TgBDP4) and α-TgIMC1 antibodies. The TgIMC1 protein served as a control, as its expression is expected to remain unchanged in the presence of IAA. The number of parasites positive for TgIMC1 (α-TgIMC1) and TgBDP4 (α-HA) was quantified from 50 randomly selected parasites. The results are presented as Mean ± SD (*n* = 3). All surviving mice were sacrificed on day 30. Relative weight loss was determined using the initial body weight recorded on the day of infection. The relevant figure illustrates the complete experimental setup and procedures involved.

### Structure predictions

The AlphaFold structure of TgBDP4 (https://alphafold.ebi.ac.uk/entry/A0A086PQW9) (64) was obtained from ToxoDB (http://toxodb.org/toxo/) (65). The disordered region was manually removed, and the amino acid sequence corresponding to residues 125 to 222, which consists of four alpha helices, was used as the input sequence for structure prediction using SWISS-MODEL (https://swissmodel.expasy.org) (40). The TgBDP4 structure was then superimposed on the PDB structure of *P*. *falciparum* BDP4 and *Homo sapiens* BRD9 (6V14) using UCSF Chimera version 1.15.

### Molecular docking and simulation

The compound structure of I-BRD9 was obtained from the PubChem database. The chemical structure was analyzed using MarvinView, an advanced viewer for 2D and 3D chemical structures. The canonical SMILES ID of the ligand I-BRD9 was recorded, and its physicochemical properties were estimated using the PubChem database. Next, using Biovia Discovery software, the selected ligands were converted into 3D structures for further analysis. The TgBDP4 protein (AlphaFold ID A0A086PQW9) was downloaded from the Protein Data Bank in PDB format. It was then purified and refined using Biovia Discovery Studio Client 2022. All unwanted co-crystals and heteroatoms were removed, and polar hydrogen bonds were added as needed to enhance binding affinity. The active binding sites were identified using the Biovia Discovery tool. A blind docking method was performed between the target protein TgBDP4 and the selected ligand I-BRD9. Molecular docking was conducted using PyRx (66), a virtual screening tool that uses the AutoDock Vina algorithm (67). This was followed by energy minimization and the conversion of PDB files to PDBQT. Furthermore, I-BRD9 was analyzed using molecular dynamics simulation to assess its stability in GROMACS 2022.2.

### Thermal shift assay (TSA)

The thermal shift assay was performed as previously described (60) using a 96-well clear low-profile plate (#MLL9601, Bio-Rad) and the CFX96 Touch Real-Time PCR detection system. Each reaction consisted of 25 μl, containing 5 μg of TgBDP4-BD and varying concentrations of I-BRD9, ranging from 5 to 20 nM. This mixture was premixed with 5 × SYPRO Orange fluorescent dye in PBS. Control wells containing only the buffer and 5 × dye were used for baseline correction. Plate was sealed with optically clear adhesive film (#MSB1001, Bio-Rad) to prevent sample loss during heating. Temperature was gradually increased by 0.5 °C every 10 s from 10 °C to 95 °C while monitoring the fluorescence intensity of the dye. Data were analysed by GraphPad Prism software and the melting point of the protein sample was determined by applying nonlinear regression through the melting Boltzmann equation: *Y* = bottom + (top - bottom)/[1 + exp(*T*_m_ - *X*/slope)].

### TgBDP4 inhibition and IC_50_ assay

The inhibitory effects of I-BRD9 (#SML1534, Sigma), (+)-JQ1 (#SML1524, Sigma), and I-BET151 (#SML0666, Sigma) on the interaction between TgBDP4-BD and H3K9ac were analyzed using MST assays. A 125 μM of His-TgBDP4-BD was incubated with serial dilutions of I-BRD9 (ranging from 1 μM to 480 PM), (+)-JQ1 (from 5 μM to 9.5 nM), or I-BET151 (from 5 μM to 39 nM), along with a FITC-labeled H3K9ac peptide (20 nM) in 10 μl of MST buffer. The reactions were incubated at 25 °C for 10 min, followed by loading the reaction mixtures into glass capillaries. Fluorescence intensity measurements and data analysis were performed as previously described. To determine the half-maximal inhibitory concentration (IC_50_), fluorescence values were measured. TgBDP4 activity without an inhibitor (control) was set to 100%. The normalised ratio of TgBDP4 activity was calculated by using the formula TgBDP4 activity (inhibitor)/TgBDP4 activity (DMSO control) × 100. The calculated relative activity (in percentage) upon inhibitor treatment was then plotted against the log-transformed concentration on the X-axis. All data were exported to GraphPad Prism. The graph was generated by selecting the 3-variable loop under the analysis option. IC_50_ values were automatically calculated and presented in the results sheet. The inhibitor experiment was repeated three times, and the results were individually plotted.

### *In vitro* assay for tachyzoite growth inhibition and EC_50_ assay

Confluent HFF monolayers in 24-well plates were infected with 10^3^ RH tachyzoites per well and incubated for 2 h at 37 °C to allow parasite invasion. Afterward, the culture medium was removed and replaced with parasite medium containing serial dilutions of I-BRD9, (+)-JQ1, or I-BET151 from 20 μM to 0.02 μM, and the plates were incubated for 5 days at 37 °C in a 5% CO_2_ incubator. Monolayers were subsequently fixed with ethanol and stained with 2% crystal violet for plaque visualization, and plaque quantification was performed as described previously. The plaque numbers from the treatment groups were normalised to the DMSO control group and plotted, with the DMSO control group considered 100%. The relative plaque formation was calculated using the formula: (Mean number of plaques in the inhibitor group/Mean number of plaques in the DMSO control group) × 100. The percentage calculation after inhibitor treatment was plotted against the log-transformed concentration on the X-axis. The entire dataset was exported to GraphPad Prism. A graph was created by selecting the three-variable loop under the analysis option, and the half-maximal effective concentrations (EC_50_) were automatically generated in the results sheet. The inhibitor experiments were repeated three times, and the results were plotted individually.

### Assays to evaluate the effect of I-BRD9 on parasite replication, invasion, and egress

The effect of I-BRD9 on parasite replication was tested by infecting coverslip-grown HFF monolayers with 10^3^ RH parasites, incubating for 1 h, and then treating with I-BRD9 at the indicated concentrations. After 36 h, the cells were processed for IFA, and parasite quantification (*n* = 3) was performed as described above.

To assess the effect of I-BRD9 on parasite invasion, RH parasites were incubated with vehicle or I-BRD9 for 1 h, then 10^3^ parasites were allowed to infect HFFs grown on glass coverslips. After 30 min, the cells were processed for IFA, and the percentage of parasite invasion (*n* = 3) was calculated as previously described.

The effect of I-BRD9 was tested on both natural and induced parasite egress. Freshly egressed RH-TIR1 or RH TgBDP4-mAID-HA parasites were inoculated on HFF monolayers. After 2 h, the medium was replaced with DMEM containing vehicle or I-BRD9. To test the effect of I-BRD9 on natural egress parasites, cells were incubated for an additional 48 h, followed by staining with antibodies α-TgGRA2 and α-TgIMC1, as described above. To test whether I-BRD9 treated parasites could egress upon calcium ionophore treatment, cells were incubated for an additional 48 h, and induced egress was initiated by calcium ionophore treatment for 2 min. Cells were then stained with antibodies α-TgGRA2 and α-TgIMC1, as described above. Fifty vacuoles were examined to count intact and collapsed vacuoles for each (*n* = 3).

### *In vitro* bradyzoite growth assays

HFF monolayers grown on glass coverslips in a 6-well plate were infected with 10^4^ ME49 tachyzoites per well and incubated for 24 h at 37 °C to allow for parasite invasion. Following this, the culture medium was removed and replaced with bradyzoite differentiation medium, which consisted of RPMI 1640 (Sigma, #R6504) supplemented with 1% FBS and 50 mM HEPES, pH 8.2. The monolayers were then treated with either the EC_50_ concentrations of I-BRD9 or DMSO vehicle and incubated for 5 days at 37 °C without CO_2_. After incubation, the infected monolayers were analyzed using IFA and stained with antibodies α-TgCST1 (58) (1:200 in PBS) and α-TgBAG1 (68) (1:200 in PBS). The number of bradyzoite cysts was determined by counting CST-stained cysts in 25 independent microscopic fields for each experiment (*n* = 3). The size of the cysts was measured by calculating the area in microns. To evaluate the viability of I-BRD9-treated bradyzoites, infected HFFs were scraped and passed through a 23-gauge needle to liberate the cysts. Subsequently, the cysts were treated with trypsin at 0.25 mg/ml for 10 min to liberate the bradyzoites. The harvested bradyzoites were then inoculated onto confluent HFF monolayers and cultured for 7 days. Afterward, Crystal violet staining was performed to detect plaque formation.

### *In vivo* efficacy of I-BRD9

To assess the effect of I-BRD9 on lethal toxoplasmosis, six-week-old male BALB/c mice were divided into five groups, with five mice in each group: 1. DMSO with infection 2. I-BRD9 at 10 mg/kg with infection 3. I-BRD9 at 20 mg/kg with infection 4. Pyrimethamine at 50 mg/kg (69) with infection 5. I-BRD9 at 20 mg/kg without infection. Four of the groups received an intraperitoneal injection of 100 RH tachyzoites. From days 2 to 12 post-infection (pi), Group 1 was given 1% DMSO *via* intraperitoneal (ip) injection, Group 2 received I-BRD9 at 10 mg/kg body weight *via* ip, Group 3 was administered I-BRD9 at 20 mg/kg body weight *via* ip, Group 4 was orally given pyrimethamine at 50 mg/kg body weight, and Group 5 served as the control group without infection (receiving I-BRD9 at 20 mg/kg body weight). All mice were weighed and monitored daily for 30 days.

## Data availability

All data are included in this publication or in the Supporting information, including figures, and tables. If additional information is desired, please contact Abhijit S. Deshmukh at abhijit@niab.org.in.

## Supporting information

This article contains supporting information.

## Conflict of interest

The authors declare that they have no conflicts of interest with the contents of this article.
