# Supplementary material for: Parasite-specific essential bromodomain protein TgBDP4 is a key epigenetic reader and a potential drug target for the parasite Toxoplasma gondii
Source: J Biol Chem. 2026 May 14;302(7):113160. doi: 10.1016/j.jbc.2026.113160 (PMC13273687; doi:10.1016/j.jbc.2026.113160)
Supplement: Supporting Figures S1 and S2, and Table S1 [file mmc1.docx]

**Supplementary Figure 1**

|  | TgBDP4 |
| --- | --- |
| HsBRD1 | 21.56 |
| HsBRD2 | 21.2 |
| HsBRD3 | 19.03 |
| HsBRD4 | 21.94 |
| HsBRD7 | 18.81 |
| HsBRD8 | 18.61 |
| HsBRD9 | 21.96 |
| HsBRD10 | 19.89 |

HsBRD10 MSVPGTPGAMEPAGEEERPPPAAEGEDDEEEVAAAAQTSGPAHGRSASSLEDADDQEEEM 60

HsBRD4 ------------------------------------------------------------ 0

HsBRD2 ------------------------------------------------------------ 0

HsBRD3 ------------------------------------------------------------ 0

HsBRD8 ----------------------------------------MATGTGKHKLLST----GPT 16

TgBDP4 ------------------------------------------------------------ 0

HsBRD1 ------------------------------------------------------------ 0

HsBRD7 ------------------------------------------------------------ 0

HsBRD9 ------------------------------------------------------------ 0

HsBRD10 EAMVIGGGCCKEQELTYELQQGYRILGEFLQEKHRGLTAPFLQPLGGVATAEEEVAEGPR 120

HsBRD4 ------------------------------------------------------------ 0

HsBRD2 ------------------------------------------------------------ 0

HsBRD3 ------------------------------------------------------------ 0

HsBRD8 EPWSIREKLCLASSVMRSGDQNWVSVSRA--------IKPFAE----------------- 51

TgBDP4 ------------------------------------------------------------ 0

HsBRD1 ---MRRKGRCHRGSAAR------------------------------------------- 14

HsBRD7 ------------------------------------------------------------ 0

HsBRD9 ------------------------------------------------------------ 0

HsBRD10 SGGRGGRAFPQQPGQGMCLLQMEEKFASGQY-----------------GGITEFVADFRL 163

HsBRD4 ------------------------------------------------------------ 0

HsBRD2 ------------------------------------------------------------ 0

HsBRD3 ------------------------------------------------------------ 0

HsBRD8 -PGRPPDWFS----QKHCASQYSELLETTETPK---------RKRGEKGEVVETVEDVIV 97

TgBDP4 ------------------------------------------------------------ 0

HsBRD1 --------HP----SSPCSVKHSPTRETLTYAQAQRMVEIEIEGRLHRISIFDPLEIILE 62

HsBRD7 ------------------------------------------------------------ 0

HsBRD9 ------------------------------------------------------------ 0

HsBRD10 MLETCYRLHGVDHWISKQGQKLE-------MML-----------------EQK------- 192

HsBRD4 ------------------------------------------------------------ 0

HsBRD2 ------------------------------------------------------------ 0

HsBRD3 ------------------------------------------------------------ 0

HsBRD8 RKLTAERVEELKKVIKETQERYRRLKRDAELIQAGHMDSRLDELCNDIATKKKLEEEEAE 157

TgBDP4 ------------------------------------------------------------ 0

HsBRD1 DDLTAQEMSECNS---------------------NKENSERPPVCLRTKRHK-----NNR 96

HsBRD7 ------------------------------------------------------------ 0

HsBRD9 ------------------------------------------------------------ 0

HsBRD10 -------LALLSRHLRE-----------------KTTIAVTSRGYYGLEDEKGTACTSTR 228

HsBRD4 ------------------------------------------------------------ 0

HsBRD2 ------------------------------------------------------------ 0

HsBRD3 ------------------------------------------------------------ 0

HsBRD8 VKRKATDAAYQARQA-----VKTPPRRLPTVMVRSPIDSASPGGDYPLGDLTPT------ 206

TgBDP4 ------------------------------------------------------------ 0

HsBRD1 VKKKN--EALPSAHGTPASASALPEPKVRIVEYSPPSAPRRPPVYYKFIEKSAE------ 148

HsBRD7 ------------------------------------------------------------ 0

HsBRD9 ------------------------------------------------------------ 0

HsBRD10 RRSTPRSLAGLTSGVFESIMVQVLRQEEQLRAKEEKRLREQERKEAEEASQKEIEEWERK 288

HsBRD4 ------------------------------------------------------------ 0

HsBRD2 ------------------------------------------------------------ 0

HsBRD3 ------------------------------------------------------------ 0

HsBRD8 ------TMEEATSGVNESEM----------------------------AVASGHLNSTGV 232

TgBDP4 ------------------------------------------------------------ 0

HsBRD1 ------ELD------NEVEY----------------------------DMDEEDYAWLEI 168

HsBRD7 ------------------------------------------------------------ 0

HsBRD9 ------------------------------------------------------------ 0

HsBRD10 LLAQAAPTCME----TM-------------------------WEIPAIGHFLCLAQ---- 315

HsBRD4 ------------------------------------------------------------ 0

HsBRD2 ----------------MLQNV----TP----HNKLPGE-----------------GN--- 16

HsBRD3 ------------------------------------------------------------ 0

HsBRD8 L--LEVGGVLPMIHGGEIQQTPNTVAA----SPAASGA-------PTLSR-LLEAGPTQF 278

TgBDP4 ------------------------------------------------------------ 0

HsBRD1 VNEKRKGDCVPAVSQSMFEFLMDRFEKESHCENQKQGEQQSLIDEDAVCC-ICMDGECQN 227

HsBRD7 ------------------------------------------------------------ 0

HsBRD9 ------------------------------------------------------------ 0

HsBRD10 ---------------QILNLPEIVFYELERCLLMPQCNAFLSKIMTSLLSPPHRRPTLHR 360

HsBRD4 -------MSAESGPGTRLRNLPVMGDGLET-SQMSTTQAQA------------------- 33

HsBRD2 -AGLLGLGPEAAAPGKRIRKPSLLYEGFES-PTMA------------------------- 49

HsBRD3 ------------------------MSTATT-VAPA------------------------- 10

HsBRD8 TTPLASFTTVASEPPVKLVPPPVESVSQATIVMMPALPAPS------------------- 319

TgBDP4 ------------------------------------------------------------ 0

HsBRD1 SN-VILFCDMCN------------LAVHQECYGVPYIPEGQ------------------- 255

HsBRD7 ------------------------------------------------------------ 0

HsBRD9 ------------------------------------------------------------ 0

HsBRD10 RPTLPYRTWEAALRQKVQQWY--TAVGQTENPDNCAEKLGLCPQFFKVLG-------EVN 411

HsBRD4 -------------------QPQPANAASTNPPPPETSNPNKPKRQTNQLQYLLRVVLKTL 74

HsBRD2 -------------------SVPALQLTPANPPPPEVSNPKKPGRVTNQLQYLHKVVMKAL 90

HsBRD3 -------------------GI-PATPGPVNPPPPEVSNPSKPGRKTNQLQYMQNVVVKTL 50

HsBRD8 ---------------SAPAVSTTESVAPVSQPDNCVPMEAVGDPHTVTVS---------- 354

TgBDP4 ------------------------------------------------------------ 0

HsBRD1 -------------------WLCRHCLQSRARPADCVLCPNKGGAFKKTDDDRWGHVVCAL 296

HsBRD7 ------------------------------------------------------------ 0

HsBRD9 ------------------------------------------------------------ 0

HsBRD10 PLEEKPFHELPFYQKVWLLKGLCDFVYETQKEVQDAVLGQPIHECREVILGYDYLENAYV 471

HsBRD4 WKHQFAW---PFQQPVDAVKLNLPDYYKIIKT--------P---MD-MGTIKKRLENNYY 119

HsBRD2 WKHQFAW---PFRQPVDAVKLGLPDYHKIIKQ--------P---MD-MGTIKRRLENNYY 135

HsBRD3 WKHQFAW---PFYQPVDAIKLNLPDYHKIIKN--------P---MD-MGTIKKRLENNYY 95

HsBRD8 --------------------------------------------MD-SSEI--------- 360

TgBDP4 ------------------------------------------------------------ 0

HsBRD1 WIPEVGFANTVFIEPIDGVRNIPPARWKLTCY--------L---CK-QKGV--------- 335

HsBRD7 ------------------------------------------------------------ 0

HsBRD9 ------------------------------------------------------------ 0

HsBRD10 HFPQFCGADVRIYKQRPFQAPEFPIPP----------------IKIQRVPRIKLEKLKCD 515

HsBRD4 WNAQECIQDFNTMFTNCYIYNKPGDDIVLMAE---ALEKLFL----QKINELPTEETEIM 172

HsBRD2 WAASECMQDFNTMFTNCYIYNKPTDDIVLMAQ---TLEKIFL----QKVASMPQEEQELV 188

HsBRD3 WSASECMQDFNTMFTNCYIYNKPTDDIVLMAQ---ALEKIFL----QKVAQMPQEEVELL 148

HsBRD8 ------SMIINSIKEECFRSGVAEAPVGSKAPSIDGKEELDLAEKMDIAVSYTGEELDFE 414

TgBDP4 ------------------------------------------------------------ 0

HsBRD1 ------GACIQCHKANCYTAFHV-----------TCAQKAGLYMKMEPVKELTGGGTTFS 378

HsBRD7 ------------------------------------------------------------ 0

HsBRD9 ------------------------------------------------------------ 0

HsBRD10 ----YVSTSNGEHRCS---RDSLPSSFK-----KEQENNFDPACCPAK------------ 551

HsBRD4 -----IVQAKGRGRGRKETGTAKPGVS---TVPNTTQASTPPQTQTPQPNPPPVQATPHP 224

HsBRD2 -----VTIPKNSHKKGAKLAALQGSVTSAHQVPAVSSVS---HTALYT------------ 228

HsBRD3 -----PPAPKGKGRKPAAGAQSA----GTQQVAAVSSVS---PATPFQSVPPTVSQTP-- 194

HsBRD8 TVGDIIAIIEDKVDDHPEVLD----VAAVEAALSFCEENDDPQSLPGPWEHPIQQERDKP 470

TgBDP4 ------------------------------------------------------------ 0

HsBRD1 -----------------------------VRKTAYCDVHTPPGCTR----RPLNI----- 400

HsBRD7 ------------------------------------------------------------ 0

HsBRD9 ------------------------------------------------------------ 0

HsBRD10 -------MILDNHDISVE----MGVKSNYEIRIRRP-----------------CEIKKTD 583

HsBRD4 FPAVTPDLIVQTP------------------------------------------VM-TV 241

HsBRD2 -----PPPEIPTT------------------------------------------VL-NI 240

HsBRD3 VIAATPVPTITAN------------------------------------------VT-SV 211

HsBRD8 VPLPAPEMTVKQERLDFEETENKGIHEL--VDIREPSAEIKVEPAEPEPVISGAEIVAGV 528

TgBDP4 ------------------------------------------------------------ 0

HsBRD1 --------------------------------------------------YGDVEMKNGV 410

HsBRD7 ------------------------------------------------------------ 0

HsBRD9 ------------------------------------------------------------ 0

HsBRD10 CCKENLEKPR----------------------------------------SPGEVTG--- 600

HsBRD4 VP-------------------------------------------------PQPLQTPPP 252

HsBRD2 PH-------------------------------------------------PSVISSPLL 251

HsBRD3 PV-------------------------------------------------PP------- 215

HsBRD8 VPATSMEPPELRSQDLDEELGSTAAGEIVEADVAIGKGDETPLTNVKTEASPESM----- 583

TgBDP4 --------------------------------------------------MYTTMSAPPS 10

HsBRD1 CRK----------------------ESSVKTVRSTSKVRKKAKKA--KKALAEPCAVLPT 446

HsBRD7 ----------------------------------MGKKHKKHKSD--K-HLYEEY----- 18

HsBRD9 ----------------------------------MGKKHKKHKAE--WRSSYEDYADK-- 22

HsBRD10 FGEPLSPG-------------------EIRFIENQEKYGEASRIKIEPSPLKENTLKSCQ 641

HsBRD4 V--PPQPQPPPAPAPQPVQSHPPIIAATPQPVKTKKG----VKRKADTT-----TPTTID 301

HsBRD2 K--SLHSAGPPLLA--------VTAAPPAQPLAKKKG----VKRKADTT-----TPTPTA 292

HsBRD3 ---AAAPPPPATPI--------VPVVPPTPPVVKKKG----VKRKADTT-----TPTTSA 255

HsBRD8 ----LSPSHGSNPIEDPLEA-----ETQ-------HKFEMSDSLKEESG-----TIF--- 619

TgBDP4 --IPLFPPPGST-----------------------AGYTFSSPAPLNSG-----GHL--- 37

HsBRD1 VCAPYIPPQRLNRIANQVAI-----QRKKQFVERAHSYWL-LKRLSRNG-----APLLRR 495

HsBRD7 ----VEKP---------------------------------LKLVLKVG-----GNEVTE 36

HsBRD9 ---PLEKP---------------------------------LKLVLKVG-----GSEVTE 41

HsBRD10 IH-VNGSHSDHPEINCHKVVRDILLEQSLQSHKKLKLTKMRAKKKK--KKKKKLKDV--- 695

HsBRD4 P--IH---E----------------PPSLPPE-------------------------PKT 315

HsBRD2 I--LA---PG-----------SPASPPGSL-E-------------------------PKA 310

HsBRD3 I--TA---SR-----------SE-SPPPLS-D-------------------------PKQ 272

HsBRD8 --------GS-----------QIKDAPGEDEE--ED-------------------GVSEA 639

TgBDP4 ---RAGGHRS-----------TLRLRPGVCS------------PVSLGSRKRPPPGSGNA 71

HsBRD1 L-------QS-----------SLQSQRSSQQR--ENDEEMKAAKEKLKYWQR-LRHDLER 534

HsBRD7 LSTGSSGHDS-----------SLFEDKNDHDK--HKDRKRKKR--KKGEKQIPGEEKGRK 81

HsBRD9 LS--GSGHDS-----------SYYDDRSDHER--ERHKEKKKKKKKKSEKEKHLDDE-ER 85

HsBRD10 ---LNENLQRKREG-LHSLAFKSYKPEIQNKLLIIKKKAKHKKHKSGKKSVSKKAITKKR 751

HsBRD4 -TKL----GQRRESS------RPVKPPKKD------VPDSQQHPAPE------------- 345

HsBRD2 ARLP----PMRRESG------RPIKPPRKD------LPDSQQQHQSS------------- 341

HsBRD3 AKVV----ARRESGG------RPIKPPKKD------LEDGEVPQHAG------------- 303

HsBRD8 AS-L--EEPKEEDQGEGYLSEMDNEPPVSE------SDDGFSIHNATLQS-------HTL 683

TgBDP4 ASALTLPTKSRRSAAQNN---VPATRSTRA------ATAAFLQHSG-------------- 108

HsBRD1 ARLLI-ELLRKREK-------LK------------------------------------- 549

HsBRD7 RRRVK-EDKKKRDR--DR---VE----NEA-------EKDLQCHAPV------------R 112

HsBRD9 RKRKE-EKKRKRER--EH---CD----TEG------EADDFDPGKKV------------E 117

...

HsBRD10 KTVIK-SPTVPEFQLICTNL-------DELRELITKIENELKDLENSRKKSGKWYHRRQA 803

HsBRD4 ----KSSKVSEQLK-CCS---------GILKEMF-----AK------KHAAYAWPFYKPV 380

HsBRD2 ----KKGKLSEQLK-HCN---------GILKELL-----SK------KHAAYAWPFYKPV 376

HsBRD3 ----KKGKLSEHLR-YCD---------SILREML-----SK------KHAAYAWPFYKPV 338

HsBRD8 ADSIPSSPASSQFS-VCSEDQEAIQAQKIWKKAIMLVWRAA------ANHRYANVFLQPV 736

TgBDP4 -ALQPSQ----------SDSASGVCTPYRVGQVLTDALNRL------QKKDKKQIFAAAV 151

HsBRD1 ------REQVK------VEQVAMELRLTPLTVLLRSVLDQL------QDKDPARIFAQPV 591

HsBRD7 LDLPPEKPLTS------SLAKQEEVEQTPLQEALNQLMRQL------QRKDPSAFFSFPV 160

HsBRD9 VEPPPDRPVRA------CRTQPAENESTPIQQLLEHFLRQL------QRKDPHGFFAFPV 165

: . .

HsBRD10 VK--ELHSTLIRLLNELLPWEPKLMKAFQRNRSRLKKDYDDFR-------------RQPD 848

HsBRD4 DVEALGLHDYCDIIKH--PMDMSTIK--SKLEAREYRDAQEFGADVRLMFSNCYKYNPPD 436

HsBRD2 DASALGLHDYHDIIKH--PMDLSTVK--RKMENRDYRDAQEFAADVRLMFSNCYKYNPPD 432

HsBRD3 DAEALELHDYHDIIKH--PMDLSTVK--RKMDGREYPDAQGFAADVRLMFSNCYKYNPPD 394

HsBRD8 TD--DIAPGYHSIVQR--PMDLSTIK--KNIENGLIRSTAEFQRDIMLMFQNAVMYNSSD 790

TgBDP4 DK--TLVPDYYVVIKE--PMFFDKMK--QKIRDRAYKTLDAFNADVELIISNCRLYNHPD 205

HsBRD1 SL--KEVPDYLDHIKH--PMDFATMR--KRLEAQGYKNLHEFEEDFDLIIDNCMKYNARD 645

HsBRD7 TD--FIAPGYSMIIKH--PMDFSTMK--EKIKNNDYQSIEELKDNFKLMCTNAMIYNKPE 214

HsBRD9 TD--AIAPGYSMIIKH--PMDFGTMK--DKIVANEYKSVTEFKADFKLMCDNAMTYNRPD 219

::. * :: . : . :

HsBRD10 HDTFNRELWTTDEGEGDLGKDSPKGEISKSIDSTEPLDILEKDHFDSDDMKLSEIDFPMA 908

HsBRD4 HEVVAMARKLQDVFEMRFAKMP--------------------------DEPEEPVVAVSS 470

HsBRD2 HDVVAMARKLQDVFEFRYAKMP--------------------------DEPLEPGPLPVS 466

HsBRD3 HEVVAMARKLQDVFEMRFAKMP--------------------------DEPVEAPALPAP 428

HsBRD8 HDVYHMAVEMQRDVL---EQIQQF-LATQLIM----------------QTSESGISAKSL 830

TgBDP4 TPYCRVAALVETCWHKLRERVKVK-FAAAAAA----------------DSADEAVVAALK 248

HsBRD1 TVFYRAAVRLRDQGGVVLRQARRE-VDS--IG----------------LEEASGMHLPER 686

HsBRD7 TIYYKAAKKLLHSGMKILSQERIQ-SLKQSID----------------FMADLQK---TR 254

HsBRD9 TVYYKLAKKILHAGFKMMSKQAAL-LGNEDTA----------------VEEPVPEVVPVQ 262

:

HsBRD10 RSKLLKKELPSKDLPKTLLKTLKRQSKQTDYVDDSTKELSPRKKAKLSTNETTVENLESD 968

HsBRD4 PA-----VPPPT-------KVVAPPSSS----D-S--------------------S---- 489

HsBRD2 TA-----MPPGL-------AKSSSESSS----EES--------------------SSESS 490

HsBRD3 AA-----PMVSK-------GAESSR----------------------------------- 441

HsBRD8 RG---------R----------DSTRKQ----DASEKDSVPMGSP------AFLLSLFMG 861

TgBDP4 PA-----------------------SSS----G----------------------SSLSG 259

HsBRD1 PA---------A----------AP-RRP----F----------------------SWEDV 700

HsBRD7 KQ---------K----------DGTDTS----Q----------------------SGEDG 269

HsBRD9 VE---------T----------AKKSKK----P----------------------SREVI 277

HsBRD10 VQIDCFSESKHTEPS------------------------FPESFASLDSVPVSTLQK--- 1001

HsBRD4 --------SDSSSDSDSSTDDSEEERAQRLAELQEQLKAVHEQLAALSQPQQNKPKKKEK 541

HsBRD2 SEEEEEEDEEDEEEEESESSDSEEERAHRLAELQEQLRAVHEQLAALSQGPISKPKRKRE 550

HsBRD3 --------SSEESSSDSGSSDSEEERATRLAELQEQLKAVHEQLAALSQAPVNKPKKKKE 493

HsBRD8 HE-WVWLDSEQDHPNDSELSN--------------DCRSLFSSWDSSLDLDVGN---WRE 903

TgBDP4 ---RGFLDPATLAQSAARVPG------------------------DS------------- 279

HsBRD1 ---DRLLDPANR----AHLGL--------E----EQLRELLDMLDLTCAMKSSG---SRS 738

HsBRD7 ---GCWQREREDSGDAEAHAF--------KSPSKENKKKDKDMLEDK--F---------- 306

HsBRD9 ---SCMFEPE---GNAC--SL-----------------------TDS--T---------- 294

HsBRD10 ---------GTKPIQALLAKNI----------------------GNKVTLTNQLPPSTGR 1030

HsBRD4 DKKEKKK------EKHKRKEEVEENKKSKAKEPPPK--KTKKNNSSNS------------ 581

HsBRD2 KKEKKKKRKAEK---HRGRAGADEDDKGPRAPRPPQPKKSKKASGSGGGSAALGPSGFGP 607

HsBRD3 KKEKEKKKKDKEKEKEKHKVKAEEEKKAKVAPPAKQAQ-QKKAPAKKANST--------- 543

HsBRD8 ---------TEDPEAEELEESS--------------------------------PE---- 918

TgBDP4 ---------ST-------PHAA---------------------SDV-GSATAGAPGGPGA 301

HsBRD1 K-----RAKLLKKEIALLRNKL---------------------SQ---QHSQPLPTG--- 766

HsBRD7 ------KSNNLEREQEQLDRIV---------------------KESGGKLTRRLVNS--Q 337

HsBRD9 ---------AEEHVLALVEHAA---------------------DEARDRINRFLPGG--K 322

HsBRD10 NALA---VEKPVLSPPEASPIKPALTCHTNTKGPLQMVYKMPCGQWLPIDLQNSSVKIQV 1087

HsBRD4 ------NVSKKEPAPMKS--KPPPTYESEEEDKCKPMSYEEKRQL--SLD-------I-- 622

HsBRD2 SGGSGTKLPKKATKTAPP--ALPTGYDSEEEEESRPMSYDEKRQL--SLD-------I-- 654

HsBRD3 -TTAGR-----QLKKGGK--QASASYDSEEEEEGLPMSYDEKRQL--SLD-------I-- 584

HsBRD8 ------REPSELLVGD---------GGSEESQE-------AARKA--SH----------- 943

TgBDP4 PGKAGVC--GDTLAGD---------GG--------------------------------- 317

HsBRD1 PGLEGFEEDGAALGPE---------AGEEV------------------------------ 787

HsBRD7 CEFERRKPDGTTT----------------------------------------------- 350

HsBRD9 MGYLKRNGDGSLL----------------------------------------------- 335

HsBRD10 QPMVDPKTGEKIMQQVLILPKNFVIQHKEGKAVAKEVPPLQQKGTEQHCSSFPQTTNINS 1147

HsBRD4 ----NKLPGEKLGRVVH------IIQSRE---------PSLKNSNPDEIE-IDFETLKPS 662

HsBRD2 ----NKLPGEKLGRVVH------IIQARE---------PSLRDSNPEEIE-IDFETLKPS 694

HsBRD3 ----NRLPGEKLGRVVH------IIQSRE---------PSLRDSNPDEIE-IDFETLKPT 624

HsBRD8 -----QNLLHFLSEVAY------LMEPLC---------ISSNESSEGCCP-PSGTRQEGR 982

TgBDP4 -----------------------------------------------SKI-VGASSKAGR 329

HsBRD1 -----------LPRLET------LLQPRK---------RS--RSTCGDSE-VEEESPGKR 818

HsBRD7 ---------------LG------LLHPVD---------PI--VGEPGYCP-VRLGMTTGR 377

HsBRD9 ---------------YS------VVNTAE---------PD--ADEEETHP-VDLSSLSSK 362

HsBRD10 SLASVF-VNSPGTVSTQLPNTAFNKTITPLSNISSARPQPLSPVTSVSNLLTPSVKTSQS 1206

HsBRD4 TL---------------------------------------------REL---------- 667

HsBRD2 TL---------------------------------------------REL---------- 699

HsBRD3 TL---------------------------------------------REL---------- 629

HsBRD8 E---------------------IKASEGE------------------RELCR-------- 995

TgBDP4 TLGAAGCVQRSDTAGKQVLGAGLGSPRDR------------------REELF-------- 363

HsBRD1 LDAGLT-NGFGGARSEQEPGGGLGRKATP------------------RRRCA-------- 851

HsBRD7 LQSGVN-TLQ-------------GFKEDK------------------RNKVT-------- 397

HsBRD9 LLPGFT-T-L-------------GFKDER------------------RNKVT-------- 381

.

HsBRD10 EAGKAKNAVSAATFSLPSASPTISSTGQPLSSTTTLNGS-----------TNPG--SSFN 1253

HsBRD4 ---------------------------------------ERYVTSCLRKKRK-------- 680

HsBRD2 ---------------------------------------ERYVLSCLRKKPR-------- 712

HsBRD3 ---------------------------------------ERYVKSCLQKKQR-------- 642

HsBRD8 ------------------ETEELSAKGDPLVAEKPLG---------ENGKPEVASAPSVI 1028

TgBDP4 ------------------L--LLRMAADPT------AAAKLEALQCLDSREQAGEAPGDS 397

HsBRD1 ------------------S--ESS----ISSSNSPLCDSSFNAPKCGRGKPALV------ 881

HsBRD7 ------------------P--VLYLNYGPYSSYAPHYDSTFANIS--------------- 422

HsBRD9 ------------------F--LSS----ATTALSMQNNSVFGDLK--------------- 402

HsBRD10 CFAQQ--------TADSSEAKQELKTVCIRDSQSILVRTRGGNTGVVKVQTNPDQ----- 1300

HsBRD4 ------------------------------------------------------------ 680

HsBRD2 ------------------------------------------------------------ 712

HsBRD3 ------------------------------------------------------------ 642

HsBRD8 CTV--------QGLLTESEEGEA------------QQESKGEDQGEVYVSEMEDQPPSGE 1068

TgBDP4 SRAEGPADATDAGLAKEAKAKET------------LKESAGPASTEVA------------ 433

HsBRD1 ---------RRHTLEDRSELISC------------IENGNYAKAARIA------------ 908

HsBRD7 --------------KDDSDLIYS----------------TYGEDSDL------------- 439

HsBRD9 --------------SDEMELLYS----------------AYGDETGV------------- 419

HsBRD10 -----NSPNTVSSSSVFTFAPQLQ--AFLVPKSTTSSSAF-----------------S-- 1334

HsBRD4 ------------------------------------------------------------ 680

HsBRD2 ------------------------------------------------------------ 712

HsBRD3 ------------------------------------------------------------ 642

HsBRD8 CDDAFNIKETPLVDTLFSHATSSKLTDLSQDDPVQDHLLFKKTLLPVWKMIASH-RFSSP 1127

TgBDP4 ----AEA---RRSSSVFGFEARQKDPFFWAPLVV-PHLLC--PPSLAWTQTLGPVEYV-- 481

HsBRD1 ----AEV----------------GQSSMWISTDAAASVLE--PLKVVWAKCSGYPSYPAL 946

HsBRD7 ------------------------------------PSDF--SIHEFLATCQDY---PYV 458

HsBRD9 ------------------------------------QCAL--SLQEFVKDAGSY---SKK 438

HsBRD10 ---P----VAGTTTTSSLSPFSQTPTSVSIPASFA-PSMGKNLKLTLGHTT--------- 1377

HsBRD4 ------------------------------------PQAEKV-DVIAG------------ 691

HsBRD2 ------------------------------------KPYTIK--KPVGKTKEELALEKKR 734

HsBRD3 ------------------------------------KPFSASGKKQAAKSKEELAQEKKK 666

HsBRD8 FLKPVSERQAPGYKD-----------------VVKRPMDLTSLKRNLSKGRIRTMAQFLR 1170

TgBDP4 ----------G--SSS----------PLSLPQKTQQPLYHKSIQAFLGEETLA-----RL 514

HsBRD1 IIDPKMPRVPGHHNGV------------TIPAP---PLDVLKIGEHMQTKSDE-----KL 986

HsBRD7 MADSLLDVLTKGGHSRTL---QEMEMSL---------PEDEGHTRTLDTAKE-------M 499

HsBRD9 VVDDLLDQITGGDHSRTL---FQLKQRRNVPMK---PPDEAKVGDTLGDSSSS-----VL 487

HsBRD10 ----------GSGDLGHVIDKTSHMPSS--PL-KSSICSSTLLPSTTSS-------SVSV 1417

HsBRD4 ------------------------------------------SSKMKGFSSS-ES-ESSS 707

HsBRD2 ELEKRLQDVSG------QLNSTKKPPKKANEKTESSSAQQVAVSRLSASSSSSDSSSSSS 788

HsBRD3 ELEKRLQDVSG------QLSSSKKPARKEKPGSAPSG----GPSRLSSSSSS-ESGSSSS 715

HsBRD8 DLMLMFQNAVMYNDSDHHVYHMAVEMRQ--EVLEQIQVLNIWLDK--------RKGSSSL 1220

TgBDP4 E--KVFPDTTR-------------------ELQKYAQHEALWAPL--------NDLR--I 543

HsBRD1 FLVLFFDNK-R-------------------------SWQ--WLPK--------------- 1003

HsBRD7 EITEVEPPG-R----------------------LDSSTQDRLIAL--------KAVT--N 526

HsBRD9 EFMSMK------------------------------SYPDVSVDI--------SMLS--S 507

HsBRD10 ISISAANFGQNNANIIHTPTKQQQVDYITKSYPVTRSEATAATNGDVISGTPVQKLMLVS 1477

HsBRD4 ESSSSDSED-SETEMA----------------PKSKKKGHPGREQKKH-HHHHHQQMQQ- 748

HsBRD2 SSSSSDTSD-SDSG---------------------------------------------- 801

HsBRD3 SGSSSDSSD-SE------------------------------------------------ 726

HsBRD8 EGEPANP----------------------------VDDGK-----------PVF------ 1235

TgBDP4 FGVDTADFPEYNSKLS-----------VDHNYLLGVGEGH-----------VQAAATLG- 580

HsBRD1 ------------SKMV----------------PLGIDETI-----------DKLKMMEG- 1023

HsBRD7 FGVPVEVFDSEEAEIF----------------QKKLDETT-----------RLLRELQE- 558

HsBRD9 LGKVKKELD---PDDS----------------HLNLDETT-----------KLLQDLHE- 536

HsBRD10 APSILSSGNGTAINMTPALTSTGVSAQKLVFINAPVPSGTSTPTLVAESLKQTLPPPLHK 1537

HsBRD4 -------------------------APAPVPQQPPPP-P------------QQPPPPP-- 768

HsBRD2 ------------------------------------------------------------ 801

HsBRD3 ------------------------------------------------------------ 726

HsBRD8 ------------------------------------------------------------ 1235

TgBDP4 -------------------------AQPPASLASPSPSP------------ESKAPSLDL 603

HsBRD1 -------------------------R-N-----------------------SSIRK---- 1030

HsBRD7 -------------------------AQNE---------R------------LSTRPPPNM 572

HsBRD9 -------------------------AQAE---------R------------GGSRPSSNL 550

HsBRD10 AYVKTPEQPQIVLI--------------PSTVGTPIKINSSPAVSQIKDVKIGLNIGQAI 1583

HsBRD4 -----PPQQQ------QQPPPPPPPPSMPQQAAPAMKSSPPPFIATQVPVLEPQLPGS-V 816

HsBRD2 ------------------------------------------------------------ 801

HsBRD3 ------------------------------------------------------------ 726

HsBRD8 ------------------------------------------------------------ 1235

TgBDP4 SCLQGLVQKQLHRRNPQRHPKPKPPGGLLARC--AAHASVASRFAALSKLSLASRAAD-I 660

HsBDP4 -AVRIAFDRAMNHLSRV-------------------HGEPT---SDLSDID--------- 1058

HsBRD7 ICLLGPSYREMHLA-----------------------EQVT---NNLKELAQQVTPGD-I 605

HsBRD9 SSLSNASERDQHHL-----------------------GSPS---R----LSVGEQPDV-T 579

HsBRD10 VNTSGTVPAIPSINILQNVTPKGEDKSSKGYILPLSTSGNSVPVSSNFVSQNITPVNESV 1643

HsBRD4 FDPIGHF-TQPIL------------------HLPQ--PELPPHLPQPPE----------- 844

HsBRD2 ------------------------------------------------------------ 801

HsBRD3 ------------------------------------------------------------ 726

HsBRD8 ------------------------------------------------------------ 1235

TgBDP4 SGGLQFS-SAPAL------------------LLAG--SEGDGTASRSFA----------- 688

HsBRD1 ------------------------------------------------------------ 1058

HsBRD7 VSTYGVR-KAMGI------------------SIP------SPVMENNFV----------- 629

HsBRD9 HDPYEFL------------------------QSP------EPAASAKT------------ 597

HsBRD10 VSSARAVNVLSVTGANLSLGSFPVTSASASAGAQPPVLVSGNDTSSRIMPI----LSNRL 1699

HsBRD4 -------------------HSTPP-HLNQHAVVSPPAL-----------HNALPQQPSRP 873

HsBRD2 ------------------------------------------------------------ 801

HsBRD3 ------------------------------------------------------------ 726

HsBRD8 ------------------------------------------------------------ 1235

TgBDP4 -------------------GQTTVARGEAEAGTSSPQVFPGREEGGRKLSAGEPLGESRG 729

HsBRD1 ------------------------------------------------------------ 1058

HsBRD7 -------------------DLTE------------------------------------- 633

HsBRD9 ------------------------------------------------------------ 597

HsBRD10 ----------------CSSSLGNTVAISTV--------------------------KTGH 1717

HsBRD4 SNRAAALPPKPARPPAVSPALTQTPLLPQPPMAQ--PPQVLLEDEEPPAPPLTSMQMQLY 931

HsBRD2 ------------------------------------------------------------ 801

HsBRD3 ------------------------------------------------------------ 726

HsBRD8 ------------------------------------------------------------ 1235

TgBDP4 DLEALHRRVEPEGADTLAPAAG-AGSIPKPGAGSREEAQLLQAA--------VRRHFDGL 780

HsBRD1 ------------------------------------------------------------ 1058

HsBRD7 ------DTEEPKKTDVA--ECG-PGGS--------------------------------- 651

HsBRD9 ------------------------------------------------------------ 597

HsBRD10 LASSVLIST-TQPVVSPKCLTSALQI--PVTVALPTPATTSPKIINTVPHSAAVPGATRS 1774

HsBRD4 LQQ-------LQKVQPPTPLLPSVKVQSQPPPPLPPPPHPSVQQQ--LQ-QQPPPPP--- 978

HsBRD2 ------------------------------------------------------------ 801

HsBRD3 ------------------------------------------------------------ 726

HsBRD8 ------------------------------------------------------------ 1235

TgBDP4 IQQVVNTSQRLVPLERRCALAPTLQCRAPGTPEVSLPRGQGPREG--SR-EAPAPAD--- 834

HsBRD1 ------------------------------------------------------------ 1058

HsBRD7 ------------------------------------------------------------ 651

HsBRD9 ------------------------------------------------------------ 597

HsBRD10 VSISKRQSRTSLQFHSPGISTTVPTNVNTNKPQTELSSLSTSPGKITNTS-NFASLPNQQ 1833

HsBRD4 --------PP-----QPQPPPQ---QQHQP---------P--PRPVHLQPMQFSTHIQQ- 1010

HsBRD2 ------------------------------------------------------------ 801

HsBRD3 ------------------------------------------------------------ 726

HsBRD8 ------------------------------------------------------------ 1235

TgBDP4 --------RKQTYLENPGAPSGLHAQRAGN---------PTDPERATVRPQLLPAKPQG- 876

HsBRD1 ------------------------------------------------------------ 1058

HsBRD7 ------------------------------------------------------------ 651

HsBRD9 ------------------------------------------------------------ 597

HsBRD10 ALVKTPSYSSAPGGTTIHTASAPSNVTSLVGSQFS------------------------- 1868

HsBRD4 P---PPPQGQ------QPPHPPPGQQPPPPQPAKPQQVIQHHHSPRHHKSDPYSTGHLRE 1061

HsBRD2 ------------------------------------------------------------ 801

HsBRD3 ------------------------------------------------------------ 726

HsBRD8 ------------------------------------------------------------ 1235

TgBDP4 P---SQETGSNSGGGISVGGGPVGNAPSAAGVQRPLGFAS------------AASAHPRE 921

HsBRD1 ------------------------------------------------------------ 1058

HsBRD7 ------------------------------------------------------------ 651

HsBRD9 ------------------------------------------------------------ 597

HsBRD10 ----------EPCIQQKIVINTSTPLAPGTQIMINGTRFIVPPQGLGAGSHVLLIS---- 1914

HsBRD4 APSP--LMIHSPQMSQFQSLTHQSPPQQN-----------VQPKKQELRAASVVQPQP-- 1106

HsBRD2 ------------------------------------------------------------ 801

HsBRD3 ------------------------------------------------------------ 726

HsBRD8 ------------------------------------------------------------ 1235

TgBDP4 KSHPPVSHALSPNASHFASLAAQPPVAPP---------RASPPSGLSTRSDSLAQVLPAS 972

HsBRD1 ------------------------------------------------------------ 1058

HsBRD7 ------------------------------------------------------------ 651

HsBRD9 ------------------------------------------------------------ 597

HsBRD10 ----TNPKYGAPLVLNSGQGIQSTPIDNSAQKITLASNNSLSGQPLQHPLRSPTKFINSF 1970

HsBRD4 LVVVK---------EEKIHSPI------------------IRSEPFSPSLRPE------- 1132

HsBRD2 ------------------------------------------------------------ 801

HsBRD3 ------------------------------------------------------------ 726

HsBRD8 ------------------------------------------------------------ 1235

TgBDP4 SHVSAKSAPHPHLVHSTLHSPQAHPVSPHSSQVRAAQPHSVSSHLLSGSFRGP------- 1025

HsBRD1 ------------------------------------------------------------ 1058

HsBRD7 ------------------------------------------------------------ 651

HsBRD9 ------------------------------------------------------------ 597

HsBRD10 GNASSIPTVHTSPQLINTTAKVPVPPPVPTVSLTSVIKSPATLLAKTSLVSAICPSNPPL 2030

HsBRD4 ------------PPKHPESIKAPVHLPQRPEMKPVDVGRPVIRPPEQN----APP--PGA 1174

HsBRD2 ------------------------------------------------------------ 801

HsBRD3 ------------------------------------------------------------ 726

HsBRD8 ------------------------------------------------------------ 1235

TgBDP4 ------------PPASTSSLSLPVPSASP--SPSSAYAQPA-----------YPP--SSL 1058

HsBRD1 ------------------------------------------------------------ 1058

HsBRD7 ------------------------------------------------------------ 651

HsBRD9 ------------------------------------------------------------ 597

HsBRD10 PSSTSVFHLD-----PPVKKLLVSPEGAILNTINTPASKVSSLSPSLSQIVVSASRSPAS 2085

HsBRD4 PDKDK-QKQEPKTPVAPKKDLKIKNMGSWASLVQK-----------HPTTPSSTAKSSSD 1222

HsBRD2 ------------------------------------------------------------ 801

HsBRD3 ------------------------------------------------------------ 726

HsBRD8 ------------------------------------------------------------ 1235

TgBDP4 PSA--------------------------------------------------------- 1061

HsBRD1 ------------------------------------------------------------ 1058

HsBRD7 ------------------------------------------------------------ 651

HsBRD9 ------------------------------------------------------------ 597

HsBRD10 VFPAFQSSGLEKPDRAAS------------------------------------------ 2103

HsBRD4 SFEQFRRAAREKEEREKALKAQAEHAEKEKERLRQERMRSREDEDALEQARRAHEEARRR 1282

HsBRD2 ------------------------------------------------------------ 801

HsBRD3 ------------------------------------------------------------ 726

HsBRD8 ------------------------------------------------------------ 1235

TgBDP4 ------------------------------------------------------------ 1061

HsBRD1 ------------------------------------------------------------ 1058

HsBRD7 ------------------------------------------------------------ 651

HsBRD9 ------------------------------------------------------------ 597

HsBRD10 ------------------------------------------------------------ 2103

HsBRD4 QEQQQQQRQEQQQQQQQQAAAVAAAATPQAQSSQPQSMLDQQRELARKREQERRRREAMA 1342

HsBRD2 ------------------------------------------------------------ 801

HsBRD3 ------------------------------------------------------------ 726

HsBRD8 ------------------------------------------------------------ 1235

TgBDP4 ------------------------------------------------------------ 1061

HsBRD1 ------------------------------------------------------------ 1058

HsBRD7 ------------------------------------------------------------ 651

HsBRD9 ------------------------------------------------------------ 597

HsBRD10 -------------------- 2103

HsBRD4 ATIDMNFQSDLLSIFEENLF 1362

HsBRD2 -------------------- 801

HsBRD3 -------------------- 726

HsBRD8 -------------------- 1235

TgBDP4 -------------------- 1061

HsBRD1 -------------------- 1058

HsBRD7 -------------------- 651

HsBRD9 -------------------- 597

**Supplementary figure 1.** An alignment of amino acid sequences of TgBDP4 and human BRD-proteins. The conserved amino acid residues are marked below with an asterisk *. Table showing the percent identity matrix.

**Supplementary Figure 2**

|  | TgBDP4 |
| --- | --- |
| HsBRD1 | 36.63 |
| HsBRD2A | 24.74 |
| HsBRD2B | 27.55 |
| HsBRD3A | 23.71 |
| HsBRD3B | 27.55 |
| HsBRD4A | 27.84 |
| HsBRD4B | 27.55 |
| HsBRD7 | 27.72 |
| HsBRD8A | 16.33 |
| HsBRD8B | 19.80 |
| HsBRD9 | 39.53 |
| HsBRD10 | 16.00 |

HsBRD1 LRLTPLT------VLLRSVLDQL--QDKDPARIFAQPVSL--KEV--------------PDYLDH 36

HsBRD2a ----RVTNQLQYL--HKVVMKAL--WKHQFAWPFRQPVDAVKLGL--------------PDYHKI 38

HsBRD2b ---GKLSEQLKHC--NGILKELLSKKHAAYAWPFYKPVDASALGL--------------HDYHDI 41

HsBRD3a ----RKTNQLQYM--QNVVVKTL--WKHQFAWPFYQPVDAIKLNL--------------PDYHKI 38

HsBRD3b ---GKLSEHLRYC--DSILREMLSKKHAAYAWPFYKPVDAEALEL--------------HDYHDI 41

HsBRD4a ----RQTNQLQYL--LRVVLKTL--WKHQFAWPFQQPVDAVKLNL--------------PDYYKI 38

HsBRD4b ---SKVSEQLKCC--SGILKEMFAKKHAAYAWPFYKPVDVEALGL--------------HDYCDI 41

HsBRD7 VEQTPLQ------EALNQLMRQL--QRKDPSAFFSFPVTD--FIA--------------PGYSMI 36

HsBRD8a -----IQAQKIWKKAIMLVWRAA--ANHRYANVFLQPVTD--DIA--------------PGYHSI 37

HsBRD8b --DDPVQDHLLFKKTLLPVWKMI--ASHRFSSPFLKPVSE--RQA--------------PGYKDV 40

HsBRD9 --STPIQ------QLLEHFLRQL--QRKDPHGFFAFPVTD--AIA--------------PGYSMI 34

HsBRD10 ---YELQ------QGYRILGEFLQEKHRGLTAPFLQPLGGVATAEEEVAEGPRSGGRGGRAFPQQ 51

TgBDP4 --PYRVG------QVLTDALNRL--QKKDKKQIFAAAVDK--TLV--------------PDYYVV 34

* : * :

HsBRD1 IKHPMDFATMRKRLEAQGYKNLHEFEEDFDLIIDNCMKYNARDTVFYRAAVRLRDQGGVVLRQA 105

HsBRD2a IKQPMDMGTIKRRLENNYYWAASECMQDFNTMFTNCYIYNKPTDDIVLMAQTLEKIFLQKVASM 107

HsBRD2b IKHPMDLSTVKRKMENRDYRDAQEFAADVRLMFSNCYKYNPPDHDVVAMARKLQDVFEFRYAKM 110

HsBRD3a IKNPMDMGTIKKRLENNYYWSASECMQDFNTMFTNCYIYNKPTDDIVLMAQALEKIFLQKVAQM 107

HsBRD3b IKHPMDLSTVKRKMDGREYPDAQGFAADVRLMFSNCYKYNPPDHEVVAMARKLQDVFEMRFAKM 110

HsBRD4a IKTPMDMGTIKKRLENNYYWNAQECIQDFNTMFTNCYIYNKPGDDIVLMAEALEKLFLQKINEL 107

HsBRD4b IKHPMDMSTIKSKLEAREYRDAQEFGADVRLMFSNCYKYNPPDHEVVAMARKLQDVFEMRFAKM 110

HsBRD8a VQRPMDLSTIKKNIENGLIRSTAEFQRDIMLMFQNAVMYNSSDHDVYHMAVEMQRDVLEQIQQF 106

HsBRD8b VKRPMDLTSLKRNLSKGRIRTMAQFLRDLMLMFQNAVMYNDSDHHVYHMAVEMRQEVLEQIQVL 109

HsBRD7 IKHPMDFSTMKEKIKNNDYQSIEELKDNFKLMCTNAMIYNKPETIYYKAAKKLLHSGMKILSQE 105

HsBRD9 IKHPMDFGTMKDKIVANEYKSVTEFKADFKLMCDNAMTYNRPDTVYY----------------- 86

HsBRD10 PGQGMCLLQMEEKFASGQYGGITEFVADFRLMLETCYRLHGVDHWISKQGQKLEMMLEQKLALL 120

TgBDP4 IKEPMFFDKMKQKIRDRAYKTLDAFNADVELIISNCRLYNHPDTPYCRVAALVETCWHKL-RE- 101

* : :. .: :. : .. *

**Supplementary figure 2.** An alignment of the bromodomain amino acid sequences of TgBDP4 and human BRD-proteins. The conserved amino acid residues are marked below with an asterisk *. Table showing the percent identity matrix.

**Supplementary Table 1**

**List of primers**

| Sr. No. | Name | Sequence |
| --- | --- | --- |
| 1 | TgBDP4_BD_F | GGAATTCCATATGGCGCTGCAGCCCTCGCA |
| 2 | TgBDP4_BD_R | CCCTCGAGGACTTTTACACGTTCTCGAAG |
| 3 | TgBDP4_mAID_F | GTTCTTCTTAGTTCCGCTTTCGTTTTAGAGCTAGAAATAGC |
| 4 | TgBDP4_mAID_R | AACTTGACATCCCCATTTAC |
| 5 | TgBDP4_MH_F | TGCGCAGCCCGCGTATCCTCCCTCTTCTCTTCCTTCTGCGGCTAGCAAGGGCTCGGGC |
| 6 | TgBDP4_MH_R | CTTCTTCCTCAAATAAAACGCTGCCGTTTTCGCCGCATGCATAGGGCGAATTGGAGCTCC |

**List of *T*. *gondii* strains**

| **No.** | **Strain** | **BEI #** | **Source/Citation** |
| --- | --- | --- | --- |
| 1 | RH-88 | NR-223 | These reagents were obtained through the NIH Biodefense and Emerging Infections Research Sources Repository, NIAID, NIH. BEI resources https://www.beiresources.org/ |
| 2 | ME49 | NR-10150 |  |
| 3 | RH-TIR1  Genotype: RHΔhxgprtΔku80; TUB1:TIR1-3FLAG, SAG1:CAT) | NR-51145 |  |
